# Defining biomaterial-driven design principles for bioabsorbable flow diverters: current state and perspectives

**DOI:** 10.1016/j.bioactmat.2026.07.045

**Published:** 2026-07-31

**Authors:** Alessandra Di Lorenzo, Amr F. Mohamed, Benedetta Isella, Daniel Behme, Alexander Loewen, Mark Vekemans, Roland Schwab, Tara C. Schmitz, Stefan Jockenhoevel, Alexander Kopp

**Affiliations:** a Fibrothelium GmbH, Triwo Technopark Aachen, Philipsstraße 8, Aachen, 52068, Germany; b Department of Biohybrid & Medical Textiles (BioTex), AME - Institute of Applied Medical Engineering, Helmholtz Institute, RWTH Aachen University, Aachen, 52074, Germany; c Embocraft GmbH, Triwo Technopark Aachen, Philipsstraße 8, Aachen, 52068, Germany; d Department of Medical Engineering, Otto-von-Guericke University of Magdeburg, Magdeburg, 39106, Germany; e Research Campus STIMULATE, Otto-von-Guericke University Magdeburg, Magdeburg, 39104, Germany; f University Clinic for Neuroradiology, Medical Faculty and University Hospital, Otto-von-Guericke-University Magdeburg, Magdeburg, 39120, Germany; g Medical Magnesium GmbH, Triwo Technopark Aachen, Philipsstraße 8, Aachen, 52068, Germany; h Meotec GmbH, Triwo Technopark Aachen, Philipsstraße 8, Aachen, 52068, Germany

**Keywords:** Bioabsorbable, Metal, Polymer, Flow diverter, Endothelialization, Bioactive coatings

## Abstract

Flow diverters (FDs) have revolutionized intracranial aneurysm management, but current permanent metallic devices remain constrained by their bulk and surface properties, which induce chronic inflammation, thrombotic risk, and impaired vessel-wall integration. In this review we address these material-driven design challenges, examining how bioactive bioabsorbable biomaterials can overcome current limitations by balancing scaffold resorption with aneurysm occlusion. Moving beyond the clinical focus of existing literature, we establish a rational design roadmap bridging the gap between bulk material properties and FD architecture. We analyze how synchronized degradation kinetics and neointimal encapsulation govern device functionality, identifying this biological isolation as a necessary safety requirement to ensure resorption occurs only after the scaffold is shielded from the active flow. We analyze the mechanical-biological trade-offs of current platforms, whereby bioabsorbable polymers offer superior flexibility but limited radial support, whereas bioabsorbable metals provide higher mechanical integrity but require precision control over degradation. Hybrid strategies, combining transient and permanent components, emerge as effective solutions to balance mechanical reliability with controlled resorption. Across material classes, sustained flow diversion and vascular healing are primarily dictated by the interplay of scaffold architecture, material composition, and time-dependent surface evolution. Our analysis highlights that converging bulk material selection with advanced interfacial engineering enables the rational design of bioabsorbable FDs that maintain temporary mechanical integrity, while ensuring predictable aneurysm occlusion. These design principles establish a scientific framework for next-generation bioactive neurovascular implants, emphasizing a materials-driven approach to optimize safety and translational potential.

## Highlights

• Design roadmap to decouple mechanical support from permanent implant presence.
• Synchronized degradation and neointimal healing defined as a core design principle.
• Mechanical trade-offs between bioabsorbable metals and polymers evaluated.
• Tiered preclinical validation pipeline mapping to biomaterial-driven criteria.
• Interfacial engineering prioritized to modulate bioactive vascular integration.

## Graphical abstract

**Figure:**
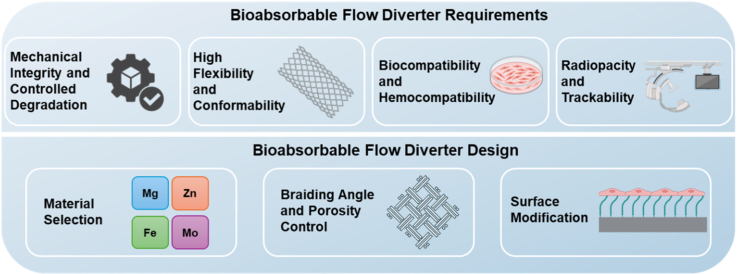

## 1 Introduction

**Fig. 1:**
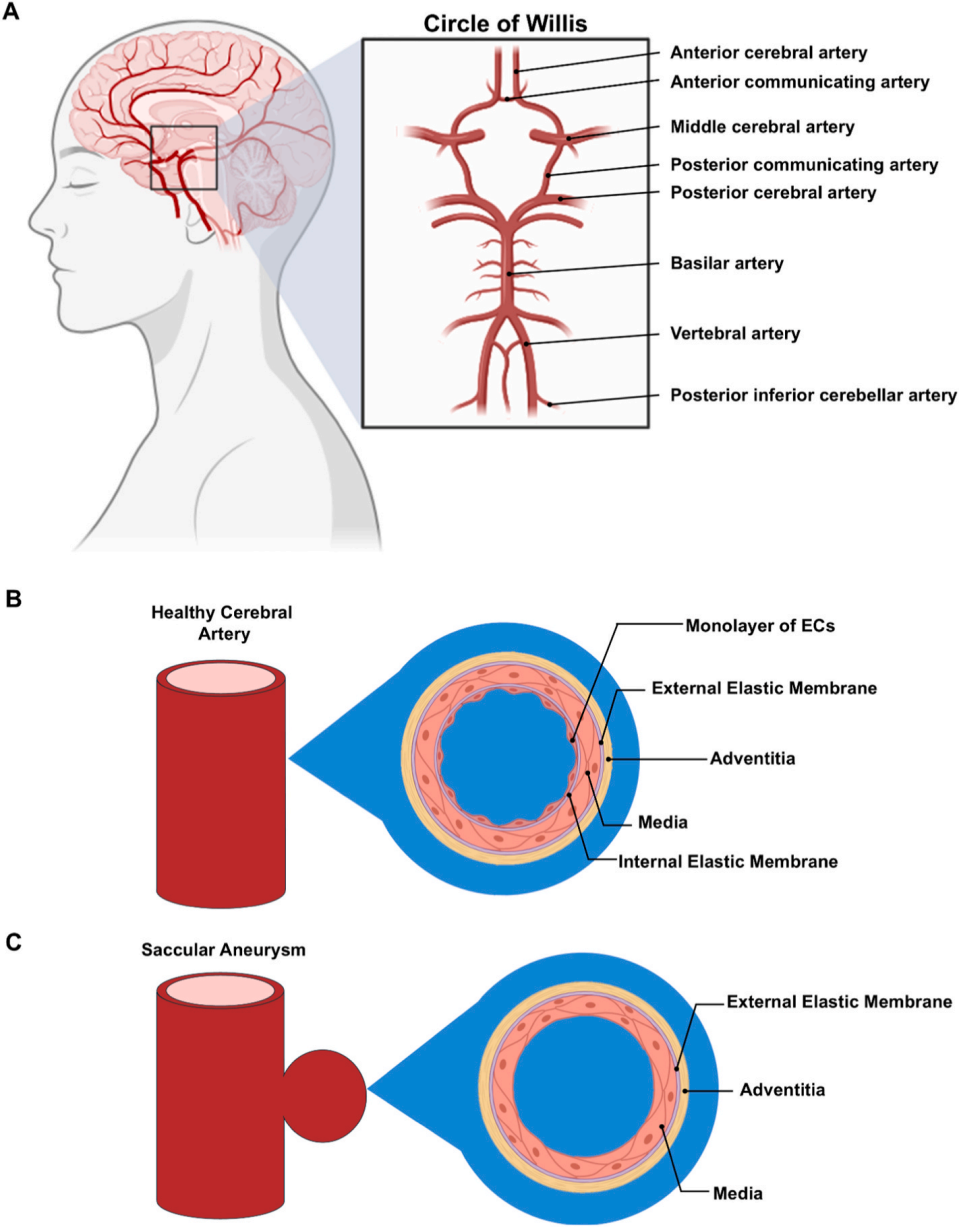
Anatomical and structural features of intracranial aneurysms. (A) Schematic representation of the Circle of Willis at the base of the human brain, (B) physiological intracranial arterial wall and (C) thinned and structurally altered wall in aneurysmal conditions.

According to a 2021 report, intracranial aneurysms affect 2–10% of the population [1]. Although most remain stable, the rupture of a subset accounts for 75–85% of non-traumatic subarachnoid hemorrhages, which are associated with high morbidity and mortality [1,2]. Depending on their size and location, aneurysms may also cause headaches or cranial nerve deficits [3,4]. They most frequently arise at arterial bifurcations within the Circle of Willis, a ring-shaped network supplying blood to the brain and surrounding tissues (Fig. 1A). Understanding the anatomical and structural features of intracranial arteries is therefore essential to contextualize aneurysm formation and progression.

Structurally, cerebral arteries are classified as small arteries. The innermost layer consists of endothelial cells, followed by the internal elastic membrane and the tunica media, which contains circumferentially aligned smooth muscle cells, elastin, and collagen fibers [5]. Finally, the adventitia is primarily composed of longitudinally oriented fibroblasts and collagen (Fig. 1B) [5].

Aneurysm-associated hemodynamic changes imply increased local wall stress, disrupted repair mechanisms, and induced intimal de-endothelialization, wall thinning, and fragmentation (Fig. 1C) [6]. In severe cases, the internal elastic membrane may be absent, a key contributor to rupture risk [6,7]. These pathological changes define a complex biological and mechanical environment that must be considered in the design of bioactive materials and endovascular devices intended to interface with the cerebral vasculature and modulate tissue responses.

**Fig. 2:**
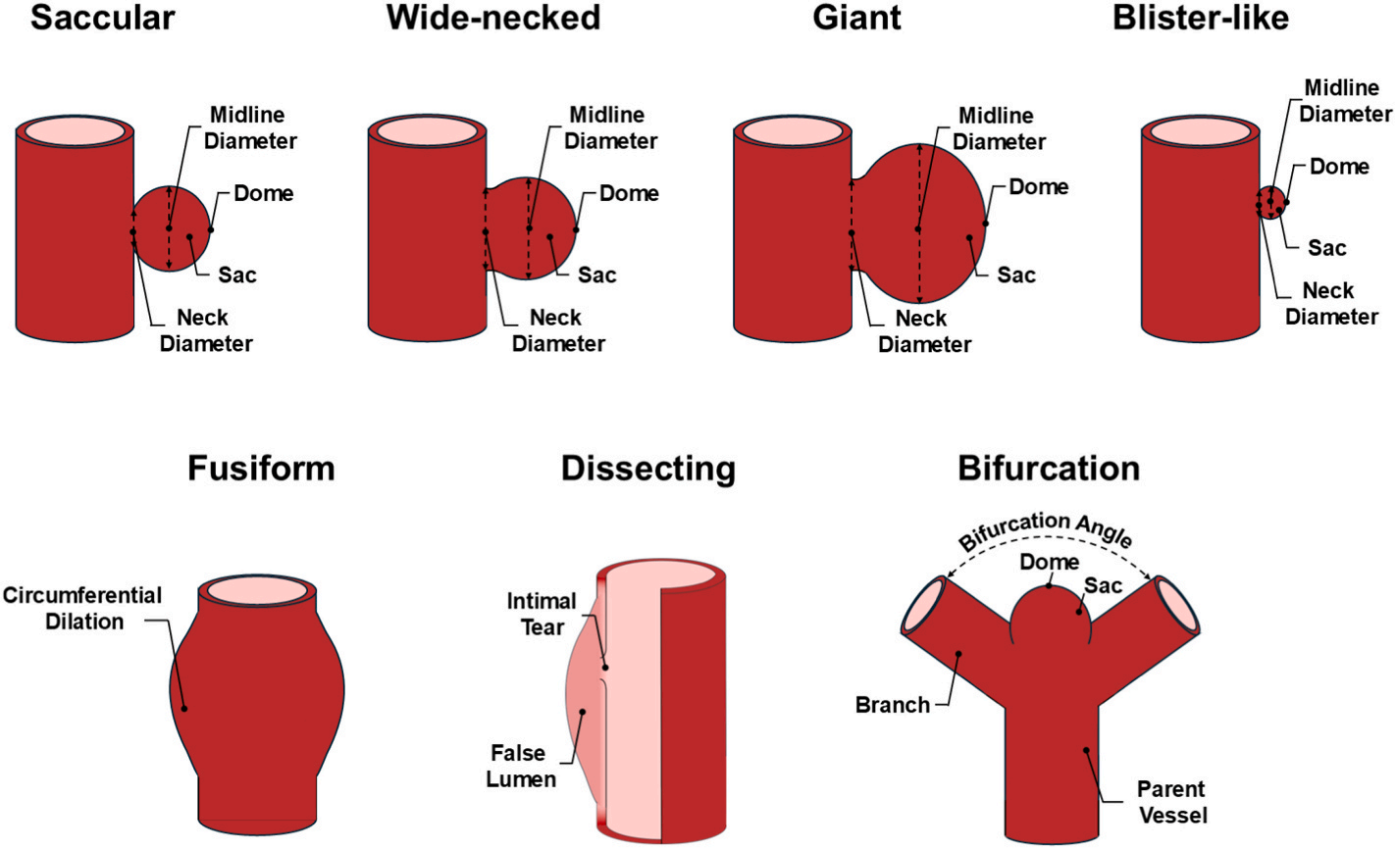
Schematic illustration of the different types of intracranial aneurysms, showing differences in shape, neck, and vessel involvement.

Aneurysms are primarily classified by shape and location [8]. The most common form is the saccular aneurysm, often found at arterial bifurcations (Fig. 2) [8]. Fusiform aneurysms, which affect the entire circumference of the artery, occur less frequently, particularly in larger vessels like the vertebral or basilar arteries [8]. Dissecting aneurysms, which occur when blood enters the vessel wall, are more common in the vertebral and carotid arteries [8]. Blister-like aneurysms, typically small and fragile, are most prone to rupture and pose particular treatment challenges, due to the marked wall fragility and high recurrence rate within a short period [9]. Treatment selection is influenced by aneurysm morphology, size, and accessibility, underscoring the need for tailored endovascular strategies [10].

Among these strategies, flow diverters have emerged as specialized stents composed of a high-density braided mesh, designed to treat intracranial aneurysms. Early flow diverter designs consisted of overlapping stents aimed at reducing aneurysm porosity and promoting intra-aneurysmal thrombosis [11,12]. Although they were not optimized for this specific application, they were able to modify intra-aneurysmal flow effectively, leading to the development of dedicated flow-diverting devices [11,12]. By promoting endothelialization, and inducing vascular remodeling, they offer a promising solution for complex aneurysms [13]. These permanent stents are now used to treat a broad range of aneurysms, from large and giant lesions to smaller ones [9,14]. However, risks remain, including thrombosis, rupture, and the requirement for prolonged dual antiplatelet therapy (DAPT) [15].

Bioabsorbable polymers and metals have emerged as candidate materials to address some of these limitations, offering the potential to reduce long-term antiplatelet dependence while enabling device designs that actively interact with the biological environment [16]. At the same time, their translation is constrained by challenges related to degradation control, mechanical integrity, and host tissue responses throughout resorption.

This review focuses on bioabsorbable flow diverters, examining their material-driven advantages in comparison with permanent devices and critically discussing the key material, preclinical, and translational challenges that currently limit their clinical implementation. By integrating advances in bioactive materials science with device engineering considerations, this work aims to provide a material-oriented framework to guide the future development of safer and more effective flow-diverting technologies.

### 1.1 Design roadmap and selection criteria

Establishing the criteria for this roadmap required an analysis of the clinical and experimental data available in current literature. We conducted a literature search across PubMed and Google Scholar for publications from 2000 through January 2026 using targeted keywords as “bioabsorbable braided stent”, “neurovascular flow diversion”, “stent endothelialization” and “flow diverter thrombogenicity”. This timeline was selected to capture both the foundational literature on permanent metallic platforms and the subsequent evolution of advanced surface coatings and bioabsorbable materials. The selected literature focuses on technical material characterizations, surface modifications and preclinical models. The review addresses safety and hemocompatibility, mechanical structural integrity, and long-term degradation kinetics, as well as translational viability regarding manufacturability and deliverability. Given the emerging nature of neurovascular-specific bioabsorbable devices, where direct clinical data are limited, these findings are supplemented with mechanistic insights and clinical evidence derived from established cardiovascular stent literature. Within this framework, we address aspects that remain unaddressed in the neurovascular literature, including the manufacturing trade-off between pre-braiding and post-braiding surface modification, the behavior of multi-material composite architectures under vascular conditions, and the synchronization between scaffold resorption kinetics and validated histopathological healing milestones.

## 2 Materials and design in flow diverters

**Fig. 3:**
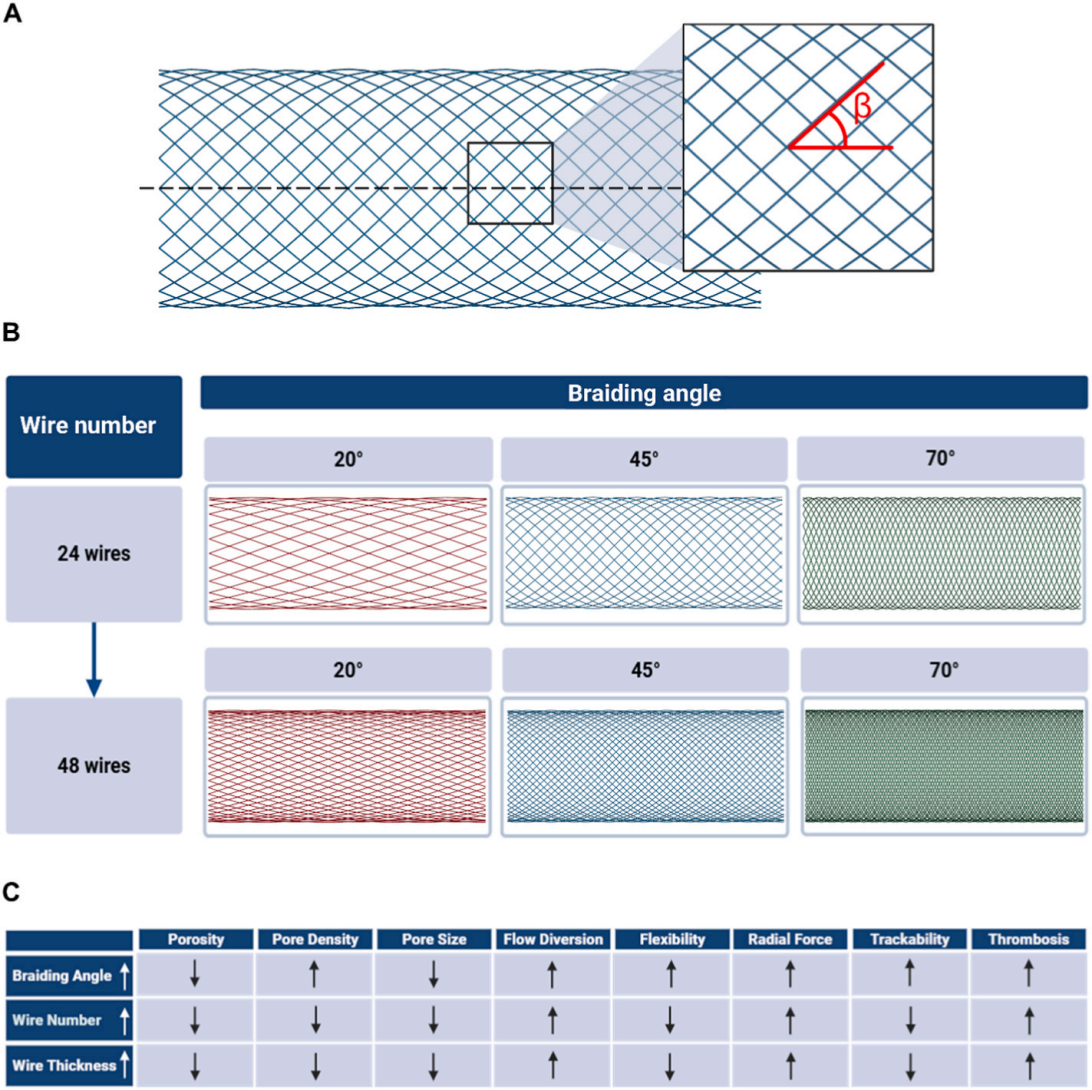
Design and geometric parameters of braided flow-diverting stents. (A) Schematic of a braided flow-diverting stent and braiding angle (β). (B) Parametric variation of braiding angle and wire number, illustrating how these parameters shape stent geometry. (C) Resulting effects of increasing braiding angle, wire number and thickness on flow diverter features.

Flow-diverting stents treat intracranial aneurysms by redirecting blood flow to induce thrombosis and endothelialization, leading to occlusion within the aneurysm [10]. They reduce intra-aneurysmal velocity, promoting aneurysm remodeling, encouraging endothelialization across the aneurysm neck, and finally restoring the physiological arterial architecture [10]. Following deployment, the drop in intra-aneurysmal flow triggers thrombus formation [13]. Within a week, shear stress decreases, isolating the aneurysm [13]. After 2–6 weeks, endothelial cells seal the aneurysm neck [13,17,18]. Over a period of 3–12 months, complete endothelialization ensures the long-term sealing of the aneurysm [18]. Ongoing research continues to refine designs and expand applications for ruptured and unruptured aneurysms, leading to the development of diverse flow diverter models tailored for different clinical needs (Fig. 3A and B) [19]. The hemodynamic performance of flow diverters is primarily determined by their geometric configuration, particularly the braiding angle and the number of constituent wires, which govern porosity, pore density and intra-aneurysmal flow diversion (Fig. 3C) [20]. Beyond these geometric considerations, additional factors contribute to overall device performance, as discussed in the following section.

An ideal flow diverter maximizes flow stagnation within the aneurysm sac while promoting rapid endothelialization across the aneurysm neck [20]. Optimized wall apposition to the parent artery supports intra-aneurysmal thrombosis and vessel healing, while minimizing the risk of thromboembolic complications or the occlusion of perforating arteries [20]. Deliverability is essential, requiring the device to navigate reliably and safely to the target site, a property determined by stent flexibility, pushability, and uniform expansion. Ensuring consistent wall apposition allows the device to conform to vessel geometry, reducing the risk of malapposition. Adequate radiopacity completes the design, enabling precise fluoroscopic guidance without compromising noninvasive follow-up imaging [20].

Although geometry governs flow modification, the choice of material is equally important. Device composition influences radial strength, fatigue resistance, and long-term stability, all of which are essential for sustained aneurysm occlusion and safe arterial reconstruction [20]. Careful consideration of both geometry and material properties is therefore central to the design of effective and reliable flow-diverting devices.

### 2.1 Commercial flow diverters

#### 2.1.1 Cobalt–chromium-based flow diverters

Cobalt–chromium (CoCr) alloys have emerged as a cornerstone in flow diverter technology due to their high elastic modulus (200–240 GPa), elevated yield strength (>2000 MPa), and excellent fatigue resistance, providing elastic strains on the order of 1% [21,22]. These properties enable thin-strut designs with high radial force, providing durable scaffolding and effective flow diversion across the aneurysm neck [[23], [24], [25], [26], [27]].

However, the intrinsic stiffness that ensures mechanical stability limits conformability in tortuous intracranial vessels. This can amplify deployment-related deformations, such as shortening, fish-mouthing, or focal braid collapse, which alter local hemodynamics and impair wall apposition [[21], [22], [23],^28^]. Furthermore, dense CoCr braiding increases the strut-to-blood contact area and local flow perturbations, promoting platelet adhesion and neointimal hyperplasia. These chronic material–tissue interactions, driven by a rigid architecture and the chemically stable Cr_2_O_3_ oxide surface, can trigger a cascade of complications (Table 1) [[24], [25], [26], [27]]. These include in-stent stenosis, thromboembolic events, and non-ischemic cerebral enhancing lesions (NICE), the latter reported with an incidence of approximately 1% [28,29]. Additionally, permanent CoCr implants may induce chronic hypersensitivity reactions to chromium or cobalt and impair long-term vascular remodeling [[30], [31], [32]]. These limitations are especially critical in pediatric patients, due to vessel growth, and elderly individuals with heightened vascular fragility. Collectively, these considerations have driven the exploration of alternative permanent metals and bioabsorbable platforms aimed at preserving flow diversion efficacy while minimizing long-term material-induced vascular perturbation [33,34].

**Table 1:** Technical specifications of commercially available flow diverters. Manufacturer, regulatory status, material composition, structural design, and deployment characteristics.

Device Name	Manufacturer	Approval Year	Material Composition	Wire number	Radiopaque Configuration	Diameter Range (mm)	Length Range (mm)	Deployment System	Key Features
Pipeline Embolization Device (PED)	Medtronic	FDA 2011	Cobalt–chromium + Platinum/Tungsten	48 braided wires	Interwoven strands	2.5–5.0	10–35	0.027–inch microcatheter	First-generation flow diverter, resheatable, effective for large aneurysms
Pipeline Flex (PED Flex)	Medtronic	FDA 2015	Cobalt–chromium + Platinum/Tungsten	48 braided wires	Interwoven strands	2.5–5.0	10–35	0.027–inch microcatheter	Highly flexible, braided design, resheatable, good vessel wall apposition
Surpass Streamline (SS-FD)	Stryker Neurovascular	FDA 2018	Cobalt–chromium + Platinum/Tungsten	48–96 wires (size-dependent)	Interwoven strands	2.5–5.3	12–50	0.027–inch microcatheter	Uniform structure, high metal coverage, designed for large and giant aneurysms
Surpass Evolve (SE-FD)	Stryker Neurovascular	FDA 2020	Cobalt–chromium + Platinum/Tungsten	48 braided wires	Interwoven strands	2.5–5.0	12–50	0.027–inch microcatheter	Uniform mesh design, improved radial force, optimized vessel conformity
Silk Flow Diverter	Balt Extrusion	CE 2009	Nitinol + Platinum	48 braided wires	Interwoven strands	2.0–5.0	10–50	0.021–inch microcatheter	Resheathable up to 90%, variable metal coverage (35–55%), flexible for tortuous anatomy
Silk + Flow Diverter	Balt Extrusion	CE 2011	Nitinol + Platinum	48 braided wires	Combined (interwoven + terminal tip + markers)	2.0–5.0	15–40	0.021–inch microcatheter	Open-cell design, flared ends for vessel apposition, increased risk of migration and thrombosis
Silk Vista Baby	Balt Extrusion	CE 2019	Nitinol + Platinum	48 braided wires	Encapsulated (Pt-core DFT wires)	1.5–3.5	10.5–26	0.017–inch microcatheter	Designed for smaller vessels, high technical success rate, low complication risk
FRED (Flow Redirection Endoluminal Device)	MicroVention	CE 2013	Nitinol + Tantalum + Platinum	48 braided wires (inner) + 16 wires (outer)	Combined (interwoven Ta + terminal Pt markers)	3.0–5.5	10–50	0.027–inch microcatheter	Dual-layer design, high radial force, resheatable up to 80%, suitable for posterior circulation
FRED Jr	MicroVention	CE 2016	Nitinol + Tantalum + Platinum	48 braided wires (inner) + 16 wires (outer)	Combined (interwoven Ta + terminal Pt markers)	<3.0	8–37	0.021–inch microcatheter	Smaller version of FRED for small intracranial arteries
DERIVO Embolization Device (DED)	Acandis	CE 2017	Nitinol + Platinum	48 braided wires	Encapsulated (Pt-core DFT wires)	3.5–6.0	15–50	0.027–inch microcatheter	Flared ends, BlueXide coating for reduced thrombogenicity, high flexibility
DERIVO mini	Acandis	CE 2018	Nitinol + Platinum	48 braided wires	Encapsulated (Pt-core DFT wires)	2.5–3.5	15–25	0.021–inch microcatheter	Tailored for smaller vessels, high visibility markers
P64 MW Flow Modulation Device	Phenox	CE 2012	Nitinol + Platinum	64 braided wires	Combined (interwoven Pt + proximal terminal markers)	2.5–5.0	9–36	0.027–inch microcatheter	Highly flexible, excellent vessel conformity, mechanical detachment system
P48 MW Flow Modulation Device	Phenox	CE 2016	Nitinol + Platinum	48 braided wires	Encapsulated (Pt-core DFT wires)	1.75–3.5	9–18	0.021–inch microcatheter	Optimized for smaller vessels, no mechanical detachment system

#### 2.1.2 Nitinol based flow diverters

Nitinol, an equiatomic nickel–titanium alloy, is widely used in flow diverters due to its corrosion resistance, biocompatibility, and unique superelastic and shape memory properties, arising from reversible thermally induced phase transformations between austenite and martensite [35,36]. Unlike linear elastic metals, elasticity in nitinol is driven by stress-induced phase transformation and can exceed 8% recoverable strain [35]. This enables braided devices to conform to tortuous cerebrovascular anatomies while maintaining uniform radial support and strut–vessel contact [35,36]. These material-driven characteristics enable complex designs, such as the dual-layer FRED family or the high-density p64/p48 braids, and support features like self-expansion and resheathing (Table 1) [35,36].

Despite these advantages, the long-term presence of permanent nitinol scaffolds is associated with significant clinical drawbacks. Acute thromboembolic events are reported in up to 5% of cases, while mechanical phenomena such as fish-mouthing, braid collapse, or braid bump can aggravate local hemodynamics and trigger hyperplastic responses [28,[37], [38], [39], [40], [41], [42]]. Even with advanced surface treatments, such as the TiO_2_-rich BlueXide coating on the DERIVO device, prolonged material–tissue interactions often contribute to delayed in-stent stenosis and neointimal hyperplasia, typically observed within 3–9 months post-implantation [30,[43], [44], [45], [46]]. Furthermore, the intrinsically low radiopacity of nitinol necessitates the integration of platinum or tantalum, increasing design complexity and influencing material–blood interactions [47,48]. Device manufacturing methods dictate how radiopaque materials are incorporated and exposed to the bloodstream, directly affecting hemocompatibility. Radiopaque elements are integrated via three main configurations. Localized terminal markers can be attached to the device ends, as seen with the platinum/tungsten markers in the FRED family. Structural strands can be interwoven within the main braid, such as the tantalum wires utilized in the FRED family or the platinum/tungsten strands in the Pipeline and Surpass devices [[49], [50], [51], [52], [53]]. Alternatively, the radiopaque component can be encapsulated within the core of drawn-filled tubing composite wires, a design feature of the platinum-core nitinol wires in the DERIVO device [54,55]. Surface-exposed materials can trigger localized galvanic coupling and uneven surface reactivity, creating heterogeneous metallic interfaces that can potentially become sites of chronic inflammation and smooth muscle cell stimulation [47]. In contrast, encapsulated configurations isolate the radiopaque material from the bloodstream, preventing direct material-blood interaction unless surface degradation occurs [42,[56], [57], [58]].

### 2.2 Bioabsorbable flow diverters

Bioabsorbable materials are designed to provide temporary mechanical support while progressively degrading to restore native vascular function [59]. An ideal bioabsorbable flow diverter would maintain sufficient mechanical integrity to promote aneurysm occlusion, while avoiding long-term implant persistence [60]. Synchronizing material degradation with vascular healing and endothelialization is therefore necessary to minimize chronic inflammation and reduce dependence on prolonged antiplatelet therapy [33]. A key safety requirement for bioabsorbable scaffolds is that pronounced degradation must be delayed until the device is fully integrated into the vessel wall.

This integration can be quantified using an established four-tier histopathological grading system for strut coverage: Grade 0 denotes bare struts, Grade 1 coverage by thrombus or fibrin without endothelium, Grade 2 a single endothelial layer without underlying smooth muscle cells, and Grade 3 endothelial coverage anchored to an underlying smooth muscle cell layer [61]. Grade 3 represents the functional benchmark, as vascular barrier integrity requires this dual-layer architecture [61,62]. Human coronary autopsy data show that Grade 3 coverage remains limited in the early post-implantation period, reaching 37.0% for durable-polymeric stents and 52.4% for biodegradable-polymeric stents at 90 days, with overall coverage exceeding 90% only at approximately 12 months [61,62]. Preclinical rabbit data corroborates this protracted timeline, although species-specific scaling must be considered: a 30-day rabbit implantation period is estimated to correspond to 6 to 12 months in humans [63]. Importantly, no equivalent pathological data exist for intracranial flow diverters, where the thinner arterial media, absence of an external elastic lamina, and different hemodynamic environment are expected to alter healing kinetics [18,64].

A precise Grade 3 threshold for safe scaffold resorption has not been established. Randomized clinical trials have demonstrated safe antiplatelet de-escalation at 1 to 3 months even though Grade 3 coverage at these timepoints remains below 50%, suggesting that histopathological coverage and thrombotic risk are not linearly related [61,62]. Moreover, the healing window varies with lesion characteristics: human autopsy data identify the acute coronary syndrome culprit site as an independent predictor of delayed coverage, and by analogy, ruptured aneurysms, wide-necked morphologies, and segments with pre-existing wall disruption would be expected to require longer scaffold persistence [62]. Defining patient-specific resorption windows will ultimately depend on prospective correlation of device-level coverage, assessed through intravascular imaging such as high-frequency optical coherence tomography, with thromboembolic outcomes. Until such data becomes available, the scaffold must retain structural integrity and radial force throughout the healing phase, limiting early mass loss and fragmentation until Grade 3 integration is achieved.

If metallic or polymeric struts break down while still exposed to the lumen, fragments could travel within the blood stream and cause embolic events. Consequently, resorption must occur intramurally, meaning inside the tissue, ensuring the scaffold is sequestered by the neointima and isolated from active flow before losing structural continuity.

**Fig. 4:**
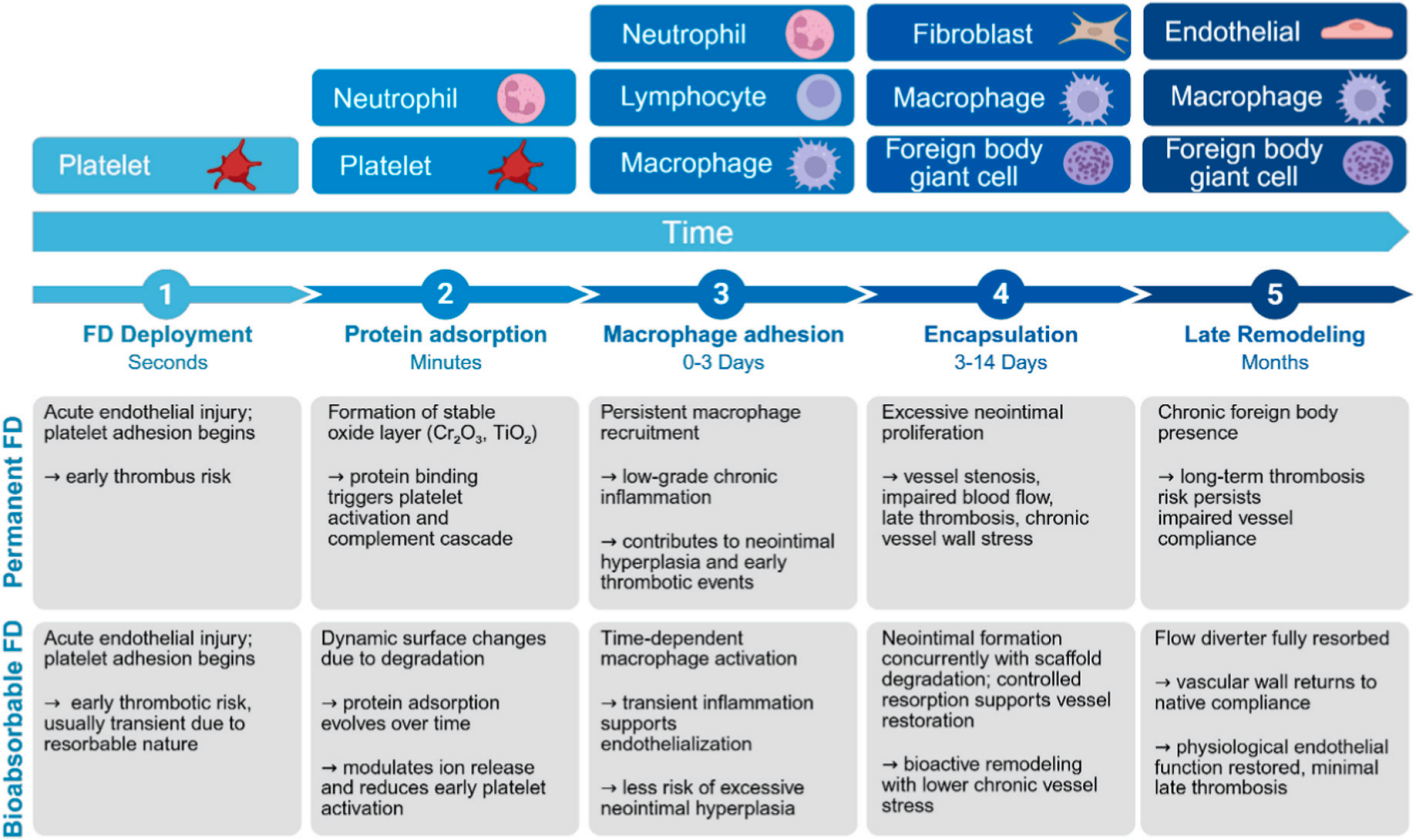
Timeline of vascular cellular responses to permanent and bioabsorbable flow diverters.

The clinical success of this transition depends on the precise synchronization between the device's degradation and the biological timeline of vessel repair. This dynamic interaction is summarized in Fig. 4, which contrasts the chronic foreign-body response of permanent implants with the staged resorption and functional restoration provided by bioabsorbable platforms.

As illustrated in the early phases of this timeline, achieving this balance requires materials that do not merely act as passive scaffolds but actively interact with the vascular environment as their properties evolve over time. In this context, bioactivity is defined as the material-driven capacity to modulate vascular healing through time-dependent changes in surface chemistry, degradation behavior, and interfacial mechanics. These dynamic material cues influence processes at the device–vessel interface, including protein adsorption, platelet activation, endothelial coverage, inflammatory signaling, and neointimal formation.

However, it is necessary to consider whether long-term stability is compromised by the eventual resorption of the scaffold, given that occlusion primarily relies on flow disruption [34]. Once endothelialization is complete, continued structural support becomes functionally redundant, suggesting that durable occlusion can be maintained independently of the scaffold [34]. The challenge remains in engineering materials with tailored degradation kinetics, ensuring sustained support throughout the healing phases without premature loss of mechanical integrity [65]. Current research addresses this challenge through the development of bioabsorbable polymeric and metallic scaffolds that balance mechanical strength, degradation rate, and biocompatibility [66]. If successfully implemented, these strategies may redefine flow diversion by mitigating the long-term limitations of permanent implants and expanding treatment paradigms for intracranial aneurysms [67]. This transition requires treating the geometric parameters that govern flow disruption, such as braid angle, wire density, and porosity, as dynamic variables rather than static constants. Because these features evolve over time according to the degradation kinetics of the material, they establish a time-dependent structure-function relationship that introduces specific mechanical and hemodynamic challenges.

#### 2.2.1 Polymer-based flow diverters

Bioabsorbable polymers have been investigated as alternative materials for flow diversion to mitigate the long-term material–tissue complications inherent to permanent metallic implants. However, their clinical viability is primarily constrained by a critical hierarchy of mechanical limitations that outweigh their biological advantages: structural bulk, compromised expansion dynamics, and the challenge of synchronized degradation.

The foremost drawback is the inherent mechanical insufficiency of polymers relative to medical-grade alloys. Compared with metallic alloys, polymers exhibit significantly lower elastic modulus, tensile strength, and radial force generation capacity. To compensate for this lack of stiffness and maintain adequate radial support during aneurysm healing, polymeric designs require markedly thicker struts or increased wire density [68]. This increased structural bulk directly influences scaffold geometry, complicating the device profile and limiting its applicability in the narrow, tortuous intracranial vasculature [67].

Compounding this structural bulk is the lack of intrinsic superelasticity and shape memory, which drastically reduces the expansion ratio and conformability of polymer-based platforms. Unlike nitinol, polymeric materials do not exhibit the recovery forces required for robust self-expansion in complex cerebrovascular anatomies. This often results in a mechanical mismatch, where differential recoil and localized malapposition create areas of incomplete vessel contact, predisposing the site to thrombus formation [60,69]. Early partially bioabsorbable designs incorporating poly (glycolic acid) (PGA) or poly-L-lactic acid (PLLA) within nitinol strands attempted to bridge this gap, but the discrepancy between the low-modulus polymer and the superelastic nitinol often resulted in non-uniform radial force distribution [60,68].

Beyond these primary mechanical hurdles, the clinical success of polymeric platforms is further dictated by the synchronization of degradation kinetics with the biological timeline of vascular remodeling. Structural integrity must be maintained well beyond the initial 6-week endothelialization window to ensure sustained healing [68]. In this context, degradation represents a complex temporal trade-off: premature bioresorption leads to the loss of flow diversion and potential recurrence, while the prolonged presence of thick-strut polymers and their acidic byproducts can trigger localized inflammatory responses or late-stage thrombotic events.

A major limitation hindering the clinical translation of bioabsorbable polymeric flow diverters is their intrinsic insufficient radiopacity, which prevents real-time fluoroscopic visualization and precise endovascular deployment. Because the low atomic number of carbon-based polymer backbones provides insufficient X-ray attenuation, unmapped device malapposition, structural twisting, or migration can occur undetected intraoperatively. To resolve this imaging challenge, current material engineering strategies focus on integrating high-atomic-number elements into the polymer architecture, though each approach introduces distinct mechanical trade-offs.

The most clinically advanced solution is the covalent incorporation of iodine directly into the polymer backbone, as implemented in the Tyrocore platform, an iodinated desaminotyrosine polycarbonate copolymer used in the Fantom bioresorbable coronary scaffold [70,71]. This design provides inherent radiopacity without relying on particulate fillers that compromise structural integrity. Alternative strategies under investigation include the deposition of high-atomic-number nanoparticle coatings or the blending of radiopaque microparticles into bioresorbable polymer matrices to enable intraoperative tracking and long-term imaging follow-up [72]. However, incorporating radiopaque additives into ultra-thin struts alters the polymer's macromolecular chain mobility and crystallinity, reducing elongation-at-break and structural ductility [72]. These effects may induce localized stress concentration sites and accelerate embrittlement, increasing the susceptibility to strut fracture during microcatheter crimping and subsequent self-expansion.

Ultimately, the clinical translation of polymer-based flow diverters depends more on overcoming fundamental limitations in radial strength and expansion ratios than on refining bioresorption chemistry alone. While preclinical studies on fully bioabsorbable PLLA devices demonstrated favorable endothelial compatibility, aneurysm occlusion rates remained lower than metallic standards (>80% at 1 year), likely due to uneven strut distribution and insufficient mechanical support during the remodeling phase [33,73]. Future directions must therefore prioritize optimized scaffold geometries that can provide metallic-like mechanical reliability within a transient, bioabsorbable framework.

#### 2.2.2 Bioabsorbable metal-based flow diverters

Bioabsorbable metal-based flow diverters are designed to combine the mechanical robustness of metallic stents with the temporary presence characteristic of degradable biomaterials, providing radial support and wall apposition during aneurysm healing while gradually resorbing to minimize long-term foreign body response. Compared with polymers, bioabsorbable metals exhibit higher elastic modulus and tensile strength, enabling improved mechanical reliability, thinner struts and subsequent reduced thrombotic risk [74]. The central design challenge across all bioabsorbable metal platforms is therefore to synchronize degradation kinetics with vascular remodeling, while maintaining structural integrity and minimizing adverse biological reactions [65].

Magnesium-based alloys are the most extensively studied bioabsorbable metals due to their biocompatibility, intrinsic antithrombotic properties, and established vascular safety profile [34]. Magnesium degrades through electrochemical corrosion, releasing Mg^2+^ ions, hydrogen gas, and hydroxide-rich surface layers. The local accumulation of hydroxide ions transiently alkalinizes the tissue, and hydrogen evolution can form microcavities, both of which lead to endothelial dysfunction and promote inflammatory responses. Degradation typically occurs within weeks, which may compromise mechanical integrity before complete aneurysm occlusion is achieved [34]. To mitigate these limitations, PLLA-coated magnesium flow diverters have been developed, delaying corrosion and preserving early structural support, while alloying with calcium, zinc, or rare earth metals can improve tensile strength and modulate degradation kinetics (Table 2) [34]. These materials are processed via precision drawing and hot extrusion to achieve the ultrafine, flexible architectures required for intracranial deployment [34,74]. To compensate for the inherently low radiopacity of magnesium, current device designs integrate auxiliary radiopaque markers in tantalum or platinum-iridium at the device extremities, ensuring reliable intraoperative visualization and long-term diagnostic follow-up without compromising the degradation profile of the structural alloy [34,74].

**Table 2:** Overview of bioresorbable biomaterials for flow diverter devices, including mechanical performance, degradation profile, and biological outcomes.

Material	Device type	Mechanical support window	Degradation/resorption timeframe	Endothelialization outcome	Main limitations	Preclinical model	Reference
PGA + nitinol (hybrid)	Partially bioabsorbable FD	Structural integrity supported by nitinol; polymer provides early porosity	PGA degradation ∼3 months	Occlusion primarily after polymer degradation	Dependence on permanent metal scaffold; delayed occlusion	Rabbit aneurysm	[68]
PLLA + nitinol (hybrid)	Partially bioabsorbable FD	Radial support dominated by nitinol	PLLA resorption months–years	Endothelial coverage heterogeneous	Mechanical mismatch PLLA–nitinol	Infra-renal rabbit aorta	[60]
PLLA	Fully bioabsorbable FD	Limited early structural support	∼1.5 years *in vitro* degradation; slower *in vivo*	Endothelial coverage improving over time	Uneven strut distribution, suboptimal occlusion	Rabbit aneurysm	[69]
PCL	Fully bioabsorbable (polymer)	Needs large struts for mechanical integrity	Slow degradation (years)	Excellent *in vitro* endothelial compatibility	Thick struts limit deliverability	*In vitro*	[67]
WE22	Fully bioabsorbable metal FD	Structural integrity ∼4–8 weeks depending on alloy	Substantial corrosion within ∼1–3 months	Early neointimal formation promoted	Rapid corrosion; premature loss of support	*In vitro*	[74]
PLLA-coated Mg	Hybrid Mg FD	Extended support vs bare Mg	Delayed corrosion compared to uncoated	Better endothelialization vs bare Mg	Limited long-term strength	Rabbit aneurysm	[34]
Fe–Mn–N	Fully bioabsorbable metal FD	Support up to ∼6 months	Accelerated corrosion vs Fe	Partial endothelialization in some cases	Early fragmentation; too rapid resorption	Rabbit abdominal aorta/elastase aneurysm	[65,75]
Fe–Mn–N/Mo	DFT wire	--	Partial at 6 months *in vivo*; FeMnN acts as sacrificial anode while Mo remains intact	Not assessed	Endothelial response not evaluated; long-term effects of Mo unknown	Murine abdominal aorta	[47]
Pure Zinc	Stent	Mechanical integrity for 3–6 months	Degradation rate <0.02 mm/year, bioabsorbed completely within 12–24 months	Confluent endothelial monolayer achieved by 6–12 months	Unvalidated intracranial	Rabbit abdominal aorta	[76]
Molybdenum	Wire	--	>6 months *in vivo*	Endothelial coverage; altered phenotype	Systemic effects of Mo unclear	Murine abdominal aorta	[77]
Mo-14Re	Wire	--	12-week *in vivo*	Rat endothelial cells adhesion and proliferation	Limited data on long-term systemic accumulation of Mo ions	Rat abdominal aorta	[78]

Building on the limitations of magnesium, iron-based alloys provide substantially higher mechanical strength, allowing thinner struts, improved wall apposition, and higher radial support [72]. Pure iron, however, degrades slowly (>1 year), producing insoluble iron oxides that can persist in the vessel wall, prolonging local inflammation and potentially delaying endothelialization. Furthermore, despite the higher atomic number of iron relative to magnesium, the ultra-fine filament diameters required for neurovascular braided configurations fail to provide sufficient X-ray attenuation, resulting in inadequate radiographic contrast under clinical fluoroscopy. To optimize the balance between strength and degradation, iron–manganese–nitrogen (FeMnN) alloys have been engineered [65,75]. Manganese accelerates corrosion through micro-galvanic effects, while nitrogen reduces the tendency to corrosion cracking as well as increases mechanical strength. FeMnN scaffolds provide structural support for up to six months *in vivo*, aligning more closely with vascular remodeling, though device fragmentation and the biological impact of iron degradation products remain under investigation [65].

Zinc-based alloys occupy an intermediate position between magnesium and iron. Zinc degrades via uniform corrosion, forming moderately stable surface layers (e.g., ZnO) and releasing metabolizable Zn^2+^ ions, which can promote endothelial proliferation and limit local inflammation [66,79]. Their *in vivo* resorption occurs over a period ranging from several months to approximately one year [80,81]. Additionally, while zinc possesses an increased atomic number compared to both magnesium and iron, yielding marginally improved X-ray attenuation, its radiopacity remains insufficient for the precise deployment verification of ultra-fine braided filaments [80]. Limited yield strength and poor creep resistance restrict their efficacy in thin filament braided structures deployed within tortuous neurovascular anatomies [81]. Because zinc-based alloys possess a comparatively low melting point relative to magnesium and iron, their homologous temperature at body temperature is sufficiently high to activate diffusion-driven creep mechanisms under sustained loading, a phenomenon that does not occur to the same extent in magnesium or iron systems under physiological conditions [81]. In self-expanding flow diverters, this behavior directly impairs the mechanical forces required for vascular reconstruction. Confinement within the delivery system causes the high bending stresses to relax during storage, converting stored elastic energy into permanent plastic deformation and preventing complete radial expansion upon release. Following deployment, the baseline chronic outward force undergoes continuous relaxation under the radial constraint of the vessel wall, a decay process further accelerated by the cyclic mechanical strain of arterial pulsation. This cumulative loss of radial support compromises long term wall apposition, increasing the risk of device migration or incomplete aneurysm occlusion [81]. Therefore, alloying with copper or silver has been explored to enhance both mechanical and corrosion properties [66].

In light of the mechanical limitations of magnesium and zinc, molybdenum (Mo) alloys have emerged as an alternative bioabsorbable metal candidate for intravascular scaffolds. Mo exhibits high yield strength, exceeding 700 MPa for pure Mo, ductility, and creep-fatigue resistance, and its standard electrode potential (∼–280 mV) supports controlled corrosion kinetics, providing structural support during the aneurysm healing while gradually resorbing [47]. The high atomic number of Mo provides inherent radiopacity, facilitating device visualization during endovascular deployment. Unlike magnesium, Mo corrosion primarily generates soluble molybdate ions (MoO_4_^2−^), which are excreted renally and release minimal hydrogen gas, thereby reducing gas-related complications [78,82]. However, the clinical translation of Mo-based devices involves complex interfacial dynamics, as the hemocompatibility profile of molybdenum is conditional and regulated by geometry, local shear environments, and the preclinical model employed. Evaluations utilizing whole blood under high shear conditions within *ex vivo* non-human primate shunt models demonstrate accelerated platelet adhesion and contact activation pathway initiation [83]. Conversely, *in vivo* studies using Mo based wires and Mo Re alloys, such as Mo 14Re, in murine arterial models have demonstrated favorable local tissue responses, showing the formation of a uniform calcium molybdenum phosphorus oxygen surface layer that supports endothelialization while limiting smooth muscle cell hyperplasia and inflammation [77,78]. *In vitro* degradation and cytocompatibility assessments further support the general biocompatibility of molybdenum under controlled conditions [82]. Despite these favorable findings, the variability of the biological response under direct blood contact remains a primary design consideration, particularly under high shear conditions. These observations align with data demonstrating that advanced processing techniques and geometric optimization alter the blood–material interface. Specifically, additively manufactured thin strut molybdenum vascular scaffolds evaluated via standardized erythrocyte lysis assays and animal vascular implantation models exhibit low hemolytic percentages and systemic tolerance [84]. Furthermore, ultrafine 50 μm molybdenum wires integrated into braided neurointerventional coil geometries exhibit low thrombogenicity indices and uniform endothelialization within vascular defects [85]. Implantation of molybdenum rhenium alloy wires within rat abdominal aorta models confirms attachment and proliferation of primary endothelial cells, although variations in endothelial cell phenotypes are reported across different murine arterial models [77,78]. Consequently, the biological performance of molybdenum under direct blood contact must be interpreted according to structural scale and the specific cellular environment rather than classified as intrinsic material incompatibility.

Geometric and mechanical scaling parameters are interconnected because the high intrinsic elastic modulus of pure molybdenum reduces compliance and anatomical conformability within tortuous cerebrovascular configurations. While the availability of ultra fine 25 to 50 μm wires addresses these geometric constraints in braided structures, the variability of the biological response under direct blood contact remains a primary design consideration. To decouple structural mechanical performance from interfacial biological interactions, composite manufacturing via drawn filled tubing (DFT) has been implemented [47].

This coaxial configuration enables the encapsulation of the resorbable radiopaque molybdenum core within an outer iron manganese nitrogen (FeMnN) sheath. This coaxial configuration isolates the structural substrate from the vascular lumen, positioning the iron alloy matrix the primary blood contacting interface to modulate initial endothelial cell migration and degradation kinetics. This design prevents immediate contact pathway activation by the underlying core under arterial shear conditions, provided the core to shell ratio is calibrated to preserve radial force, flexibility, and fatigue resistance [47]. Importantly, utilizing an ultra-fine core (<20 μm) serves as a critical safety mechanism when the outer iron-based sheath eventually resorbs. Because the core diameter is so small, the total mass of molybdenum exposed to the bloodstream is drastically restricted. This micro-scale geometry prevents a massive, localized release of corrosion products, thereby mitigating the conditional thrombogenicity of bare molybdenum and ensuring a safe, low-volume final degradation phase.

Despite these architectural developments, a substantial discrepancy remains between isolated filament characterization and the multiaxial stress states characteristic of neurovascular flow diversion. The extant literature regarding the biological and mechanical performance of molybdenum relies predominantly on unbraided wire implantations, peripheral stenting configurations, or auxiliary neurointerventional devices rather than operational braided flow-diverting meshes [77,84,85]. Braided flow-diverting geometries impose specific mechanobiological demands, including high structural mesh densities, cyclic multiaxial flexing, and localized hemodynamic stagnation within the aneurysm dome, parameters that remain unvalidated for molybdenum-based configurations. Additionally, the metabolic clearance pathways of downstream degradation products introduce toxicological uncertainties. Although the outer sheath delays substrate exposure, eventual core dissolution yields soluble molybdate anions whose long-term clearance profiles and potential cytopathic effects within the renal, hepatic, and central nervous systems require comprehensive preclinical quantification before clinical implementation can be considered.

Across all bioabsorbable metal platforms, the trade-off between mechanical support and resorption kinetics necessitates careful optimization. Excessive material burden, through increased strut number, thickness, or density to compensate for lower strength, can increase thromboembolic risk, and influence endothelialization [86]. Consequently, optimization of alloy composition, surface coatings, and scaffold geometry is essential to achieve temporary yet effective flow diversion, maintaining support during aneurysm healing while avoiding long-term constraints of permanent metallic implants. The clinical translation of Mo-based platforms will depend on demonstrating that the DFT architecture can deliver the mechanical and biological performance observed in isolated filament studies within a fully braided, intracranial flow-diverting configuration, a validation that remains to be achieved.

### 2.3 Current state of surface modifications

**Fig. 5:**
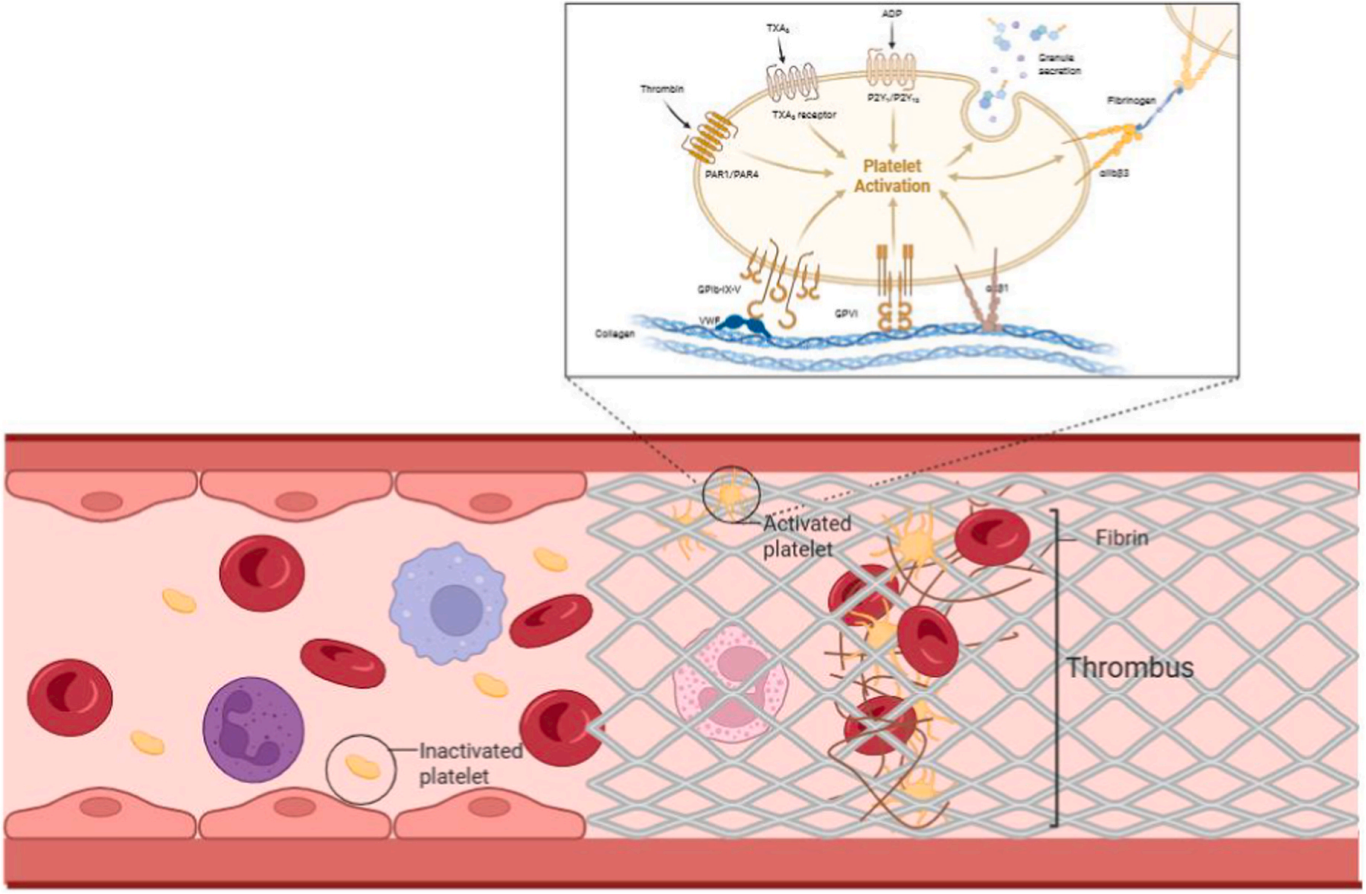
Platelet adhesion and thrombogenesis at the vascular interface. The illustration details the biomolecular mediators of clot formation, from initial platelet adhesion to the subendothelial matrix (via GPIb-IX-V and GPVI) to agonist-driven activation (Thrombin, ADP, TXA_2_) and final thrombus stabilization within a fibrin scaffold.

The need for dual antiplatelet therapy (DAPT) is a major limitation of current flow-diverting stents, and it is typically aspirin combined with clopidogrel, which act on complementary platelet activation pathways. Aspirin inhibits thromboxane A_2_-mediated platelet aggregation, while clopidogrel blocks adenosine diphosphate (ADP)-induced platelet activation, thereby reducing thrombus formation on the device surface. This pharmacological requirement arises from the intrinsically thrombogenic interaction between circulating blood components and the metallic stent surface, which promotes platelet adhesion and activation [31,57,87]. The passive oxide layers present on cobalt-chromium and nitinol flow diverters promote early protein adsorption and platelet adhesion at the blood-device interface (Fig. 5). Although progressive endothelial coverage may partially shield the metallic surface over time, the underlying material chemistry remains unchanged, sustaining a material-driven propensity for platelet interaction [31,57,87].

Consequently, prolonged DAPT is required to mitigate the risk of acute and subacute thrombotic complications [31,57,87]. However, this necessity reflects an intrinsic limitation of current materials rather than optimal biological integration, and is associated with an increased risk of hemorrhagic complications, including intracranial and systemic bleeding events [31,57,87,88]. This reliance on DAPT significantly restricts the applicability of flow diverters in ruptured aneurysms, where intensified coagulation activity and bleeding risk make prolonged antiplatelet therapy undesirable [31,57,87,88].

Beyond direct blood–material contact, vascular remodeling involves complex cell-cell interactions. Activated platelets release platelet-derived growth factor (PDGF) and transforming growth factor-β (TGF-β), which stimulate smooth muscle cell (SMC) migration and proliferation, while these migrating SMCs express tissue factor and collagen matrices, sustaining local thrombin generation. Additionally, macrophage subpopulations actively regulate immunothrombosis at the tissue-device interface. While the M1 phenotype promotes localized pro-inflammatory cascades, M2 macrophages secrete nitric oxide (NO) and prostaglandin I2 (PGI2) to suppress platelet activation, concurrently expressing CD39 to enzymatically degrade luminal ADP and limit downstream thrombotic signaling (Fig. 5) [89,90].

Furthermore, red blood cells (RBCs) influence biomaterial thrombogenicity through coupled mechanical and biochemical mechanisms. In arterial flow, deformable RBCs migrate toward the vessel centerline, pushing platelets outward through repeated collisions and concentrating them in the plasma-rich layer near the stent struts. Furthermore, hemolysis within stagnant intra-aneurysmal flow fields releases ADP, triggering localized platelet signaling pathways, while RBCs entrapped within the fibrin mesh transition into polyhedrocytes that restrict plasmin diffusion and delay endogenous fibrinolysis, thereby stabilizing the thrombus [91,92].

At the strut-blood interface, this intrinsic surface reactivity, combined with local flow disturbances induced by device geometry, creates a complex microenvironment that challenges the achievement of prompt endothelialization, particularly when migrating SMCs are actively responding to the mechanical trauma of deployment [90,93].

Under physiological conditions, endothelial cells (ECs) rest on a basement membrane supported by an underlying layer of contractile SMCs, rather than directly on synthetic surfaces [18]. Consequently, because flow-diverting devices do not rely on local antiproliferative agents to restrict SMC activity, prompt endothelialization depends on modulating the early signaling cascade to support cell migration while preventing a transition into a hyperproliferative state [18,93]. Advanced surface modifications resolve this challenge by altering plasma protein adsorption dynamics to establish a non-thrombogenic, provisional matrix rich in proteins such as fibronectin [18,64]. This layer serves as a provisional ECM that bypasses the immediate requirement of an underlying, mature SMC substrate, actively supporting direct ECs adhesion and rapid monolayer formation over the device struts while stabilizing the adjacent medial tissue response [18,64].

Building on this approach, current strategies have focused on surface modifications more than on the transition to bioresorbable materials. Advanced surface coatings for commercially available flow-diverting devices can improve hemocompatibility, reduce thrombogenicity, and accelerate endothelialization without compromising mechanical performance. Strategies include coatings that modulate protein adsorption and platelet adhesion, polymeric or inorganic layers that influence interfacial hydration and flow, and drug-eluting surfaces that locally suppress platelet activation while supporting vascular healing [94,95] These modifications maintain intra-aneurysmal thrombosis while limiting parent-vessel thrombogenicity, enhancing both safety and long-term device performance.

**Fig. 6:**
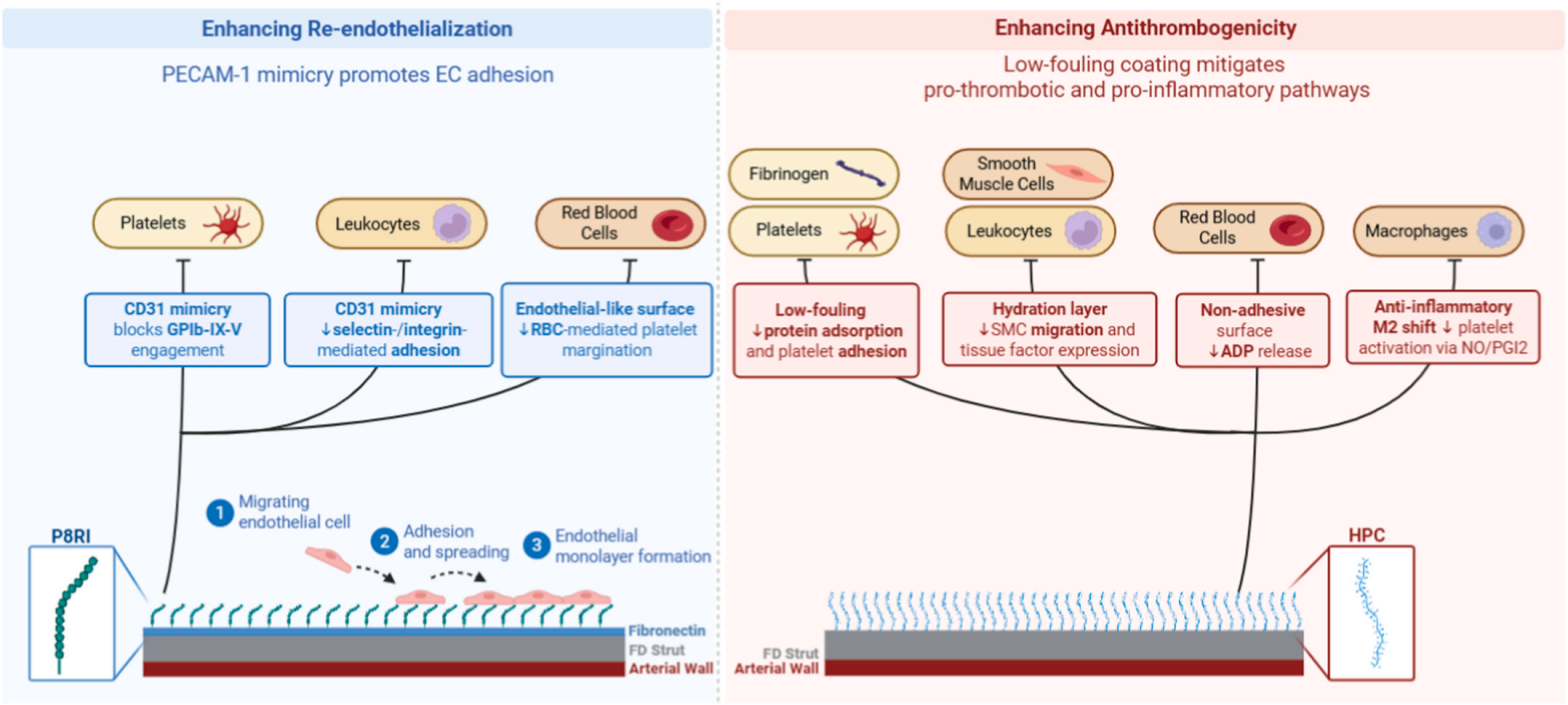
Coating strategies for flow diverters. Promoting re-endothelialization versus enhancing antithrombogenicity.

Among the technologies already implemented in clinical practice, CD31-mimetic coatings, such as the P8RI peptide functionalized on the Silk Vista Baby, enhance endothelial adhesion and reduce platelet activation by mimicking PECAM-1, a key regulator of vascular homeostasis (Fig. 6) [94]. Preclinical studies showed that endothelial coverage improved while thrombogenic responses decrease without affecting vessel patency [94]. However, the long-term clinical efficacy of such coatings and their impact on reducing ischemic complications remain under investigation [94].

Building on this concept of biomimicry, clinically established coating strategies aim to passivate the blood-device interface while supporting vascular integration through different material mechanisms. One such example is fibrin-heparin coatings, such as HEAL technology on the DERIVO 2heal embolization device (Acandis) [96]. This dual-layer nanocoating passivates the implant by binding heparin to a fibrin matrix, reducing platelet adhesion while promoting rapid endothelialization [96]. Preclinical studies have demonstrated significant thromboresistance with intact endothelial coverage, and the ongoing Clinical Safety and Efficacy of the DERIVO 2heal Embolization Device (REheal) trial is evaluating its clinical potential into a safe shortening of the DAPT regimen in human cohorts [96].

This coating architecture also illustrates how acute thromboresistance and long-term endothelialization function as complementary rather than opposing objectives. Surface-immobilized heparin retains its catalytic activity for antithrombin III, providing immediate, localized anticoagulation from the first moment of blood contact [97,98]. Because heparin is covalently bound to the fibrin matrix rather than systemically administered, this antithrombogenic activity is confined to the device-blood interface without systemic bleeding risk, directly suppressing thrombin generation and platelet adhesion during the critical early post-implantation window [97]. Simultaneously, the underlying fibrin network functions as a provisional biomimetic scaffold that bypasses the initial phases of protein adsorption and thromboinflammation, providing structural cues for endothelial cell adhesion and neointimal formation [98]. As mature endothelialization progresses over the initial months post-implantation, the early heparin-mediated antithrombogenic protection acts as a critical temporal bridge until the autologous endothelial monolayer assumes this role on a sustained physiological basis [61,62,95].

Beyond peptide- and protein-based approaches, polymeric and phospholipid-mimetic coatings represent another major class of surface modifications designed to regulate blood–material interactions. Phosphorylcholine-based coatings, such as Medtronic's Shield technology, introduce a biomimetic phospholipid layer that reduces platelet receptor engagement, prevents fibrinogen adsorption, and stabilizes plasma proteins in a non-activating conformation, thereby limiting platelet activation and supporting uniform endothelial coverage along the stent struts [99]. *In vitro* studies report decreased platelet deposition and reduced P-selectin expression, indicating improved thromboresistance at the material–tissue interface.

Similarly, clinically available glycan-based hydrophilic polymer coatings (HPC), applied to p48 and p64 flow diverters, mimic the endothelial glycocalyx by presenting negatively charged, hydrophilic sugar moieties that reduce protein adsorption, repel platelets, and guide early endothelial migration over the device struts [100]. Notably, among coated flow diverters currently in clinical use, HPC-coated devices have generated the most advanced clinical evidence supporting antiplatelet simplification, although whether safe single antiplatelet therapy with aspirin alone can serve as the definitive clinical standard remains to be demonstrated in randomized controlled trials [100]. Poly (2-methoxyethyl acrylate) (PMEA)-based coatings, such as X-technology on FRED-X devices, establish a stable hydration layer at the blood–polymer interface, preventing conformational changes of adsorbed fibrinogen and von Willebrand factor. This mechanism reduces platelet adhesion and thrombin generation under flow, enhancing thromboresistance while facilitating endothelial coverage along the device [101]. Collectively, these surface modification strategies improve the hemocompatibility of flow diverters by regulating early protein adsorption, platelet adhesion, and subsequent cellular responses at the blood–device interface [93,95,[98], [99], [100]]. Rather than targeting acute thromboresistance and long-term endothelialization as isolated or opposing mechanisms, these coating designs treat them as complementary stages of a continuous vascular healing cascade [95].

As the device integrates, the coating passivates the foreign surface until a mature, fully functional endothelial layer develops. Once established, this autologous endothelial lining provides sustained, long-term antithrombotic protection and guides progressive vessel remodeling [95]. Nevertheless, challenges remain regarding long-term durability, thrombogenicity, and vessel remodeling [95]. Additionally, their ability to fully eliminate the need for antiplatelet therapy is still under investigation [95]. Validation of reduced material thrombogenicity relies primarily on *in vitro* platelet activation assays, fibrinogen adsorption kinetics, and short-term animal histopathology. Consequently, clinical antiplatelet de-escalation protocols remain guided by single-arm registries and cohort studies rather than randomized trials.

#### 2.3.1 New coating approaches and active functionalization

Current research is shifting the paradigm from passive surface passivation toward active bio-functionalization of the vascular microenvironment. Rather than merely shielding the metallic surface, these next-generation strategies aim to orchestrate a pro-healing response by targeting three distinct biological pathways: enzymatic modulation, biophysical cell-guidance, and the selective recruitment of endogenous progenitors.

The first methodology utilizes enzymatic pathways to replicate the natural antithrombotic signaling of the vascular wall. In line with this concept, a nanoscale coating was developed by using recombinant human thrombomodulin, which can be deposited on commercial flow diverters [102]. Both *in vivo* and *in vitro* studies demonstrated enhanced hemocompatibility compared to bare devices, paving the way for the integration of synthetic or natural polymers to further optimize surface performance [102].

Parallel to biochemical signaling, a second approach targets accelerated healing through biophysical guidance to eliminate the reliance on spontaneous cell migration. This is exemplified using superparamagnetic iron oxide nanoparticles (SPIONs) to enhance endothelial cell adhesion [103]. In a rabbit model, autologous blood outgrowth endothelial cells (BOECs) were labeled with SPIONs and injected into the arterial lumen after device deployment [103]. The application of an external magnetic field caused these magnetically responsive cells to adhere to the surface of the magnetized stents, facilitating endothelialization, but the small diameter of flow diverter wires limits the ability to achieve sufficient magnetization for clinical translation [103].

A third strategy harnesses the body's endogenous regenerative potential by capturing circulating endothelial colony-forming cells (ECFCs) directly from the bloodstream [104]. In this approach, anti-CD144 antibodies recognizing ECFCs were conjugated to an RRGW linker peptide and immobilized on aminated surfaces [104]. Tested under controlled shear stress in a parallel-plate flow chamber, these functionalized substrates effectively captured circulating ECFCs, confirming that antibody-based recruitment is a viable route for creating self-healing vascular implants [104].

While these next-generation active bio-functionalization strategies demonstrate reproducible endothelial integration and acute thromboresistance within preclinical frameworks, their capacity to safely reduce clinical antiplatelet dependence remains unproven. The vast majority of current data derive from *in vitro* assays or induced animal aneurysm models lacking preexisting vascular pathology, evaluated almost exclusively on stable, permanent metallic substrates. Consequently, these experimental models omit the altered hemodynamics, chronic inflammatory signaling, and impaired endothelial kinetics characteristic of diseased human neurovascular remodeling. The lack of standardized testing protocols that combine physiological shear stress with evolving interfacial degradation kinetics remains a primary material barrier. This data disparity prevents the direct translation of these surface modifications into validated, predictable clinical protocols for antiplatelet de-escalation. Bridging this translational gap requires a fundamental understanding of the immediate bio-molecular events that govern the blood-biomaterial boundary layer upon implantation.

Upon exposure to the bloodstream, metallic biomaterial surfaces are immediately coated by plasma proteins, forming a dynamic protein layer that influences corrosion behavior, ion release and subsequent cell–material interactions, including endothelialization and platelet activation [105]. Protein adsorption on biomaterials is an early event that strongly influences subsequent corrosion behavior and biological responses. In degradable metals, this adsorption both affects and is affected by surface chemistry evolution, whereas in passivated permanent metals the protein layer forms on a relatively stable oxide surface (e.g., Cr_2_O_3_ and TiO_2_) [106]. On these surfaces, protein adsorption is primarily dictated by the static oxide chemistry and any engineered coatings, leading to relatively constant patterns of blood activation and cell adhesion. In this context, the primary function of coatings on durable stents is strictly limited to ensuring thrombo- and hemocompatibility. Consequently, permanent metals present a quasi-static surface whose bioactivity is largely defined by these specific engineered modifications. In contrast, the evolving surface chemistry of bioabsorbable metals establishes a time-dependent bioactive interface that continuously reshapes protein binding, platelet interactions, and endothelial responses. Therefore, surface strategies for bioabsorbable flow diverters must achieve a dual functional objective: they must simultaneously provide hemocompatibility and serve as a barrier to control the degradation kinetics of the underlying metal matrix. This dual-action approach is essential to prevent premature mechanical loss while managing the biological response during the critical stages of vascular remodeling.

**Fig. 7:**
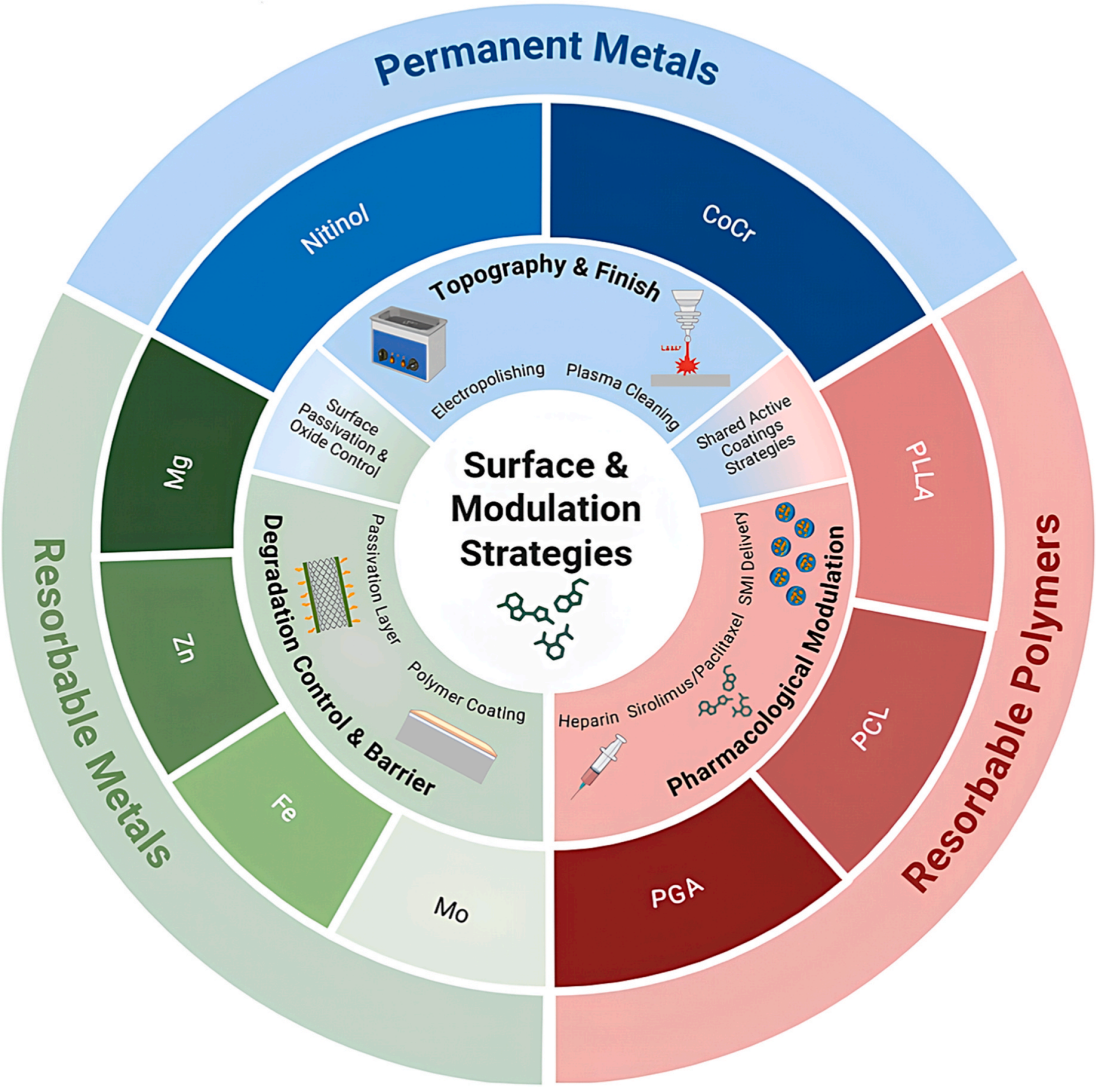
Materials for next-generation flow diverters. Overview of biomaterials combined with advanced surface-modification techniques aimed at improving device biocompatibility, endothelialization, and long-term performance.

Advances in alloy design, surface engineering, and manufacturing techniques are therefore needed to balance mechanical performance with controlled resorption [59]. Additional strategies include polymeric or nanostructured coatings to modulate these degradation-endothelialization trade-offs, as well as advanced manufacturing techniques, such as precision additive manufacturing, to optimize strut geometry and mechanical integrity. Various surface treatments can be applied, such as laser surface modification, plasma treatment and electropolishing, to reduce device thrombogenicity (Fig. 7) [86].

Pharmacologically active small molecules originally investigated for neuroprotection in cerebrovascular diseases [107,108] or specifically targeting pathological signaling cascades in intracranial aneurysms represent promising candidates for incorporation into flow diverter coating platforms [108]. Current research focuses on incorporating clinically-validated compounds directly into coatings. Following the model of cardiovascular drug-eluting stents, SMIs can alleviate the specific drawbacks of metallic scaffolds: for instance, Nrf2 pathway agonists (e.g., Dimethyl fumarate) or HMGB1 inhibitors can target oxidative stress and the inflammatory microenvironment at the aneurysm site [107,108]. Simultaneously, established agents such as Sirolimus or Heparin can be adapted to prevent intimal hyperplasia and acute thrombosis. This synergy allows the coating to regulate degradation while the SMIs ensure predictable vascular remodeling, overcoming the limitations that often hinder the clinical progression of newly developed molecules [107,108].

However, whether these surface functionalization and drug-eluting strategies can truly reduce systemic DAPT dependence remains unproven due to a reliance on indirect benchmarks. Specifically, thrombomodulin and anti-CD144/RRGW coatings are validated only via static *in vitro* assays or parallel-plate flow chambers, while SPION-mediated cell guidance has demonstrated short-term endothelialization exclusively in healthy rabbit models lacking native neurovascular pathology [[102], [103], [104]]. Although clinical data from permanent platforms coated with a stable 0.5 μm thick hydrophilic phosphorylcholine bilayer show safe DAPT discontinuation at 6 months, confirmed by Grade 3 neointimal coverage via angioscopy, multi-center registries indicate that current real-world protocols remain highly empirical: 59% of clinicians maintain DAPT for 6 months, 33% for 3 months, and 83% mandate an immediate switch to potent P2Y_12_ inhibitors like Prasugrel or Ticagrelor in cases of clopidogrel resistance [109].

These rigid clinical timelines cannot be extrapolated to dynamically degrading surfaces due to a fundamental spatiotemporal mismatch at the blood-biomaterial interface. For absorbable substrates, coatings routinely fail to remain functional during mass loss. As a degradable magnesium matrix corrodes, galvanic or pitting processes generate localized hydrogen gas evolution and severe hydroxide-induced pH spikes exceeding 8.5 *in vitro*, which destroy coating-substrate adhesion [110]. Conversely, aliphatic polyester substrates undergo autocatalytic bulk hydrolysis, trapping acidic oligomers that drop the local pH below 5.0 [111]. These acute microenvironmental alterations trigger premature polymer delamination, micro-cracking, or uncontrolled drug dumping within 14 to 30 days post-implantation, paradoxically exposing a highly pro-thrombotic degrading core to the bloodstream before functional tissue integration occurs.

While the primary objective of bioabsorbable flow diverters is to eliminate the permanent metallic substrate associated with late thromboembolic complications, an immediate reduction in antithrombotic dependence cannot be definitively assumed. Translational data derived from first-generation coronary bioresorbable devices demonstrate that elevated strut thicknesses exceeding 150 μm, combined with non-uniform degradation kinetics, disrupt local hemodynamics by creating micro-vortices and areas of low endothelial shear stress [[112], [113], [114]]. This significantly increased the incidence of very late scaffold thrombosis, clinically necessitating the intensification of antiplatelet regimens utilizing potent P2Y_12_ inhibitors rather than protocol de-escalation [114]. Consequently, true clinical antiplatelet de-escalation for absorbable flow diverters remains unsubstantiated until surface engineering achieves a proven, synchronized degradation profile between the conversion layer, the functional topcoat, and the underlying structural matrix under physiological shear stress.

Although bioabsorbable stents are already routine in the field of clinical cardiovascular medicine, their application in neurointervention remains largely experimental. While press releases have reported first-in-human implantations of polymer-based neurovascular flow-diverting stents (ReSolv), no publicly registered large-scale clinical trials of fully bioabsorbable flow diverters are currently listed on ClinicalTrials.gov, indicating that the clinical translation of such devices is still in its early stages [60]. Both magnesium-based and polymeric bioabsorbable coronary implants have reached the market, though the first-generation Absorb BVS (Abbott Vascular) was withdrawn in 2017 due to elevated rates of very late scaffold thrombosis [114]. Other polymeric platforms including FORTITUDE (Amaranth Medical, Mountain View, CA), DESolve (Elixir Medical Corporation, Sunnyvale, CA), MeRes100 (Meril Life Sciences, Vapi, India), XINSORB (Shandong HuaAn Biotechnology, China), and ReZolve (Reva Medical, San Diego, CA) show variable performance influenced by strut thickness, degradation kinetics, and drug elution, with consequences for restenosis, neointimal hyperplasia, and thrombosis [115]. These experiences underscore the importance of material selection, precise fabrication, and surface modification, offering lessons that may guide the design and eventual clinical translation of bioabsorbable flow diverters for neurovascular applications [112,113].

## 3 Hurdles in the development of biodegradable flow diverters

### 3.1 Material-driven challenges in bioabsorbable flow diverters

We previously examined the intrinsic material properties governing degradation kinetics, mechanical behavior, and surface bioactivity, but the clinical translation of bioabsorbable flow diverters introduces additional challenges that stem from their dynamic evolution within the intracranial environment. Unlike permanent metallic devices, whose structure remains relatively stable over time, bioabsorbable flow diverters continuously change in mechanical integrity, surface chemistry, and structural continuity during degradation. This temporal evolution creates a moving interface between device, blood, and vessel wall, with implications that extend beyond initial implantation performance [34].

#### 3.1.1 Degradation–endothelialization mismatch

For bioabsorbable materials, the therapeutic window is defined by the temporal overlap between mechanical support and endothelial maturation [34]. A material that loses structural continuity before stable neointimal coverage is established risks re-exposure of the aneurysm neck to flow. Conversely, delayed resorption prolongs foreign body presence and chronic inflammatory stimulation [34,69].

Different material classes illustrate this timing sensitivity. Rapidly corroding metals may reduce scaffold integrity within weeks if not surface-modified, whereas slowly hydrolyzing polymers or slowly absorbing alloys may remain structurally present long after endothelialization. Importantly, degradation in braided devices may not occur uniformly: local variations in strain, braid angle, or surface finishing can produce heterogeneous resorption patterns, potentially altering porosity and flow modulation over time [34,69].

Furthermore, this therapeutic window varies according to the anatomical and pathological phenotype of the lesion [8,18]. In configurations such as small, unruptured saccular aneurysms, device incorporation typically follows predictable healing kinetics [18,64]. Conversely, complex geometries involving large, wide-necked, or fusiform aneurysms introduce significant hemodynamic alterations, as high-velocity blood flow disrupts the local stasis required for thrombosis and exerts elevated shear stresses that impair endothelial cell migration, thereby requiring extended mechanical persistence of the scaffold [7,17,18,53]. In addition to geometry, the baseline clinical presentation governs the underlying biological timeline of vascular repair [62].

Quantitative histopathological evidence from human autopsy series demonstrates that acute, pro-inflammatory vascular environments impair these reparative mechanisms, acting as independent predictors of diminished endothelialization within the first year post-implantation, specifically within 370 days or less [62]. Under these acute conditions, treated lesions exhibit a higher prevalence of delayed tissue incorporation, frequently presenting with Grade 0 or Grade 1 classification, whereas chronic, stable clinical profiles demonstrate higher rates of Grade 3 coverage within shorter timeframes [61,62].

Consequently, in acute pathological contexts, the device's degradation kinetics must be appropriately prolonged to prevent early structural degradation or localized, byproduct-associated cytotoxicity before complete cellular sequestration is achieved. Thus, the key challenge is ensuring that degradation kinetics, spatial uniformity, and mechanical persistence are aligned with the biological timeline of intracranial aneurysm healing.

#### 3.1.2 Mechanical integrity during degradation

Unlike permanent nitinol or cobalt–chromium scaffolds, bioabsorbable flow diverters progressively lose cross-sectional area and material continuity. This induces a time-dependent structure-function relationship where progressive architectural modifications span from microstructural degradation to macrostructural failure. As degradation advances, reductions in radial force, fatigue resistance, and structural coherence may occur simultaneously [69].

Polymeric scaffolds, due to their lower intrinsic modulus, often require increased strut dimensions to achieve adequate initial radial support. During hydrolytic breakdown, fluid absorption causes the random scission of polymer chains within the matrix. This continuous reduction in molecular weight alters crystallinity and material ductility without an immediate loss of mass or structural geometry, thereby maintaining temporary macrostructural stability while progressively reducing radial strength long before mass loss becomes apparent, which can further reduce mechanical stability [72,111]. Metallic bioabsorbable systems, although initially stronger, may experience localized weakening at corrosion sites, undergoing surface-driven electrochemical dissolution characterized by rapid wire thinning. This localized degradation accelerates material loss particularly at wire intersections where mechanical stress concentrations are elevated [65,74,110]. To modulate these kinetics and preserve early structural integrity, protective polymer coatings can be applied to delay fluid ingress, reduce localized galvanic activity, and ensure the structural framework remains intact, minimizing the risk of micro-fracture or fragmentation under cyclic cerebrovascular loading [34].

In the intracranial circulation, characterized by small vessel diameter and high tortuosity, even minor reductions in flexibility or localized loss of braid integrity may influence wall apposition. As the wire cross-sectional area decreases, the attenuated radial force induces braid-angle drift, shifting the orientation of interwoven wires due to reduced friction at junctions. This architectural shift directly modifies local pore density and porosity; mesh expansion or distortion increases local pore size, potentially allowing higher volumetric flow into the aneurysm neck [20,28]. If degradation kinetics outpace tissue healing, the loss of wall apposition creates gaps at the endothelial interface, altering local wall shear stress and destabilizing the hemodynamic environment [34,69]. Therefore, mechanical testing for bioabsorbable flow diverters must incorporate time-dependent evaluation under physiologically relevant loading conditions. Notably, unlike permanent nitinol or cobalt–chromium implants, these devices do not require extreme fatigue life since they do not need to endure pulsation over years.

To validate performance during the resorption phase, researchers utilize pulsatile flow loops with silicone vascular replicas *in vitro*. Furthermore, preclinical *in vivo* models are essential to monitor how degradation affects hemodynamics and vessel wall apposition during the therapeutic window, requiring precise synchronization between device degradation and neointimal formation [116]. *In vivo*, bioabsorbable scaffolds elicit a transient local inflammatory response that drives early endothelialization and neointimal coverage across the aneurysm neck. Protective coatings or specific alloy compositions can optimize this window, promoting complete aneurysm occlusion and the establishment of a mature, collagen-rich neointimal layer consistent with Grade 3 strut coverage before advanced wire fragmentation occurs, ensuring long-term vascular stability after complete device resorption [61]. This wire fragmentation poses distinct mechanical risks in hybrid braided configurations combining bioabsorbable structural alloys, such as FeMnN, with permanent radiopaque filaments. Evolving electrochemical corrosion of the degradable metal matrix eliminates the geometric constraints maintaining the braided assembly, triggering an abrupt release of the stored elastic strain energy within the non-degradable strands. In hybrid designs utilizing tantalum, this mechanism causes the permanent wires to recoil and project outward beyond the nominal device boundary. This macrostructural failure mode, the harpoon effect, leads to vessel puncture or wire migration. Notably, because this behavior depends strictly on the elastic modulus, yield strength, and manufacturing of the radiopaque element, alternative materials with different mechanical profiles, such as nitinol, may not exhibit identical deformation patterns during scaffold degradation [47,75].

#### 3.1.3 Thrombogenicity vs surface bioactivity

The interaction between blood and device surface is dynamically altered in degradable systems. As polymers hydrolyze or metals corrode, surface chemistry, roughness, and ionic microenvironment continuously change. These evolving surfaces modulate protein adsorption profiles, platelet activation pathways, and endothelial cell adhesion in a time-dependent manner.

Increased luminal surface exposure, whether due to thicker polymeric struts, higher wire density required for mechanical compensation, or transient irregularities during corrosion, may elevate thrombogenic susceptibility, particularly during intermediate stages of degradation. Moreover, material degradation during incomplete endothelialization drives a chronic thromboinflammatory response sustained by evolving surface chemistry [74,117].

Unlike single-material devices, drawn filled tubing (DFT) systems introduce sequential material interfaces driven by different degradation kinetics [47]. In FeMnN/Mo DFT configurations, the outer iron alloy sheath undergoes more rapid degradation compared to the slower-corroding molybdenum core, which is utilized for its mechanical performance and radiopacity. As discussed in Section 2.2.2, the hemocompatibility of molybdenum is conditional and model-dependent, with favorable responses reported in murine implantation models and additively manufactured scaffolds but accelerated platelet adhesion observed under high shear whole-blood conditions [77,78,83,84]. If the outer sheath dissolution rate outpaces neointimal maturation, premature exposure of the underlying molybdenum core occurs within an unhealed vascular tract.

Consistent with the challenges inherent to permanent devices, the integration of radiopaque elements establishes heterogeneous material interfaces prone to electrochemical potential disparities [47]. In bioabsorbable scaffolds, this condition is amplified because the divergent corrosion kinetics of the structural alloy and the radiopaque additives promote non-uniform surface degradation. This spatio-temporal inconsistency in material dissolution continuously modulates protein adsorption and platelet activation, thereby complicating the thrombogenic profile relative to devices of homogeneous composition [83]. Therefore, regardless of whether radiopacity is provided by discrete markers, supplementary filaments, or internal core materials, engineering priorities must focus on maintaining interfacial stability to preclude the premature exposure of internal components, which might otherwise catalyze thrombus formation before endothelial cell coverage is established. This transition introduces a distinct hemocompatibility challenge in braided architectures, where the high localized metal density at the device neck combined with low velocity flow stagnation within the aneurysm dome promotes accelerated clotting if core exposure occurs prior to complete endothelial coverage [83].

Thus, in bioabsorbable devices, thrombogenicity is not a static material property, but a temporally evolving characteristic driven by material chemistry and degradation behavior [114]. Surface modification strategies effective for permanent alloys cannot be directly extrapolated, as degradable substrates continuously regenerate new interfaces. Effective design requires surface passivation or protective coatings to regulate initial hydrolysis or prevent a burst of ion release, ensuring a stable transition toward endothelialization.

#### 3.1.4 Potential accumulation of degradation products in the brain

Given their location within the cranium and in close proximity to the brain, degradation products from bioabsorbable flow diverters may cross the blood–brain barrier (BBB), so it is important to ensure they do not reach neurotoxic concentrations. To the best of our knowledge, no specific studies on the neurotoxicity of bioabsorbable flow diverters have been conducted *in vivo*, and neurotoxicity has not been considered as part of biocompatibility testing [118]. The BBB is a highly selective barrier, restricting most molecules and particles and posing a considerable challenge even for nanoparticles engineered to deliver drugs to the brain [119]. However, BBB permeability increases after ischemic or hemorrhagic stroke, which could alter the local concentration and distribution of degradation products [120,121]. Safety assessments must distinguish between the chemical effects of ion accumulation and the physical risk posed by migrating degradation fragments, which could mechanically obstruct the vessels.

Toxicology studies indicate that several metallic elements relevant to bioabsorbable devices are biologically essential yet can become neurotoxic following the disruption of redox balance, oxidative stress and interference with metal homeostasis. From adjacent fields, it is known that magnesium and zinc can cross the BBB and may act as neuroprotective agents with generally low neurotoxicity at physiological concentrations, but excessive Zn^2+^ levels can induce excitotoxicity, oxidative stress, and neuronal apoptosis [122]. While precise thresholds for neurotoxicity in the context of zinc-based flow diverters are not yet established, monitoring systemic and local zinc concentrations will be critical for safety assessment. Similarly, iron may also accumulate in the brain, but this can lead to the accumulation of reactive oxygen species and has been associated with neurodegenerative diseases [123,124]. A similar picture emerges for copper [125]. A more detailed review of bioabsorbable flow diverters prepared from these three elements is available elsewhere [126]. Few specific studies have been conducted on the neurological effects of common alloying elements. Yttrium seems unable to cross the BBB, whereas other rare earth elements, including neodymium, can accumulate in the brain and affect brain function [122,127]. Similarly, for molybdenum, preliminary *in vivo* studies report favorable acute biocompatibility and no overt renal toxicity [85,128], but systematic central nervous system safety data remain scarce. While it is a biologically essential element, general neurotoxicological evidence indicates that excessive molybdenum can induce neurotoxicity via oxidative stress and neuroinflammation. Consequently, evaluating its local degradation kinetics and potential accumulation is critical to ensure concentration remains within safe homeostatic thresholds.

Bioabsorbable polymer-based flow diverters release degradation products, such as lactic acid, glycolic acid, and their oligomers from PLLA, PGA, or PCL scaffolds, which may accumulate in the brain over time. The long-term consequences of these polymeric byproducts on neural tissue are unexplored [129,130]. Adverse events in the brain stemming from bioabsorbable flow diverters must be addressed by future research, including biocompatibility testing, the accumulation of degradation products in the brain, standard systemic toxicity testing, and animal studies focusing on behavioral indicators.

Beyond the potential neurotoxicity of degradation products, insufficient endothelial coverage during device resorption may expose the metallic or polymeric struts to circulating blood, increasing the risk of thrombus formation and distal embolization [131]. Additionally, premature strut fragmentation may lead to thromboembolic or ischemic complications. To ensure the safety of next-generation bioabsorbable flow diverters, preclinical evaluation should therefore not only assess the systemic and neurological toxicity of degradation products, but also the mechanical integrity and fragmentation behavior of the device during degradation and resorption. Future *in vivo* studies should therefore assess both biochemical and mechanical safety parameters to ensure complete and stable vessel healing during the device lifetime.

### 3.2 Lack of standardized pre-clinical animal models

There are currently no standard animal models for the investigation of flow diversion interventions in aneurysm treatment. *In vivo* studies tend to use mouse, rat or rabbit models, whose hemodynamic and healing timeframe differs from human patients [34,132,133]. Each model captures different individual aspects of aneurysm healing, but no unifying model fully replicates human vascular responses to flow diverters [115]. Rodents, rabbits, canines, and swine have all been used to study flow diversion, each providing distinct advantages in terms of anatomical similarity, procedural feasibility, and hemodynamic relevance (Table 3) [134]. Since no single animal model can fully capture the complex biological and hemodynamic environment of human intracranial aneurysms, a sequential, multi-stage testing approach is required to establish translational predictability and overcome current preclinical limitations. Small-animal screening using murine and rodent models constitutes the first tier of this pathway. These models offer specific logistical advantages, including high reproducibility, cost effectiveness, and a broad availability of molecular markers. They provide early material or coating assessments and deliver deep insights into the biology of arterial aneurysms far beyond what can be achieved in large animals [132,133]. They are highly valuable for evaluating the early vascular tissue response, assessing systemic biocompatibility, and predicting how the physiological hemodynamic environment affects the degradation rates of metals or polymers [77,78]. Since the small caliber of rodent arteries severely restricts device size and limits endovascular deployment in terms of trackability, early screening can utilize alternative material configurations, such as wires implanted into the vessel lumen [77,78].

**Table 3:**
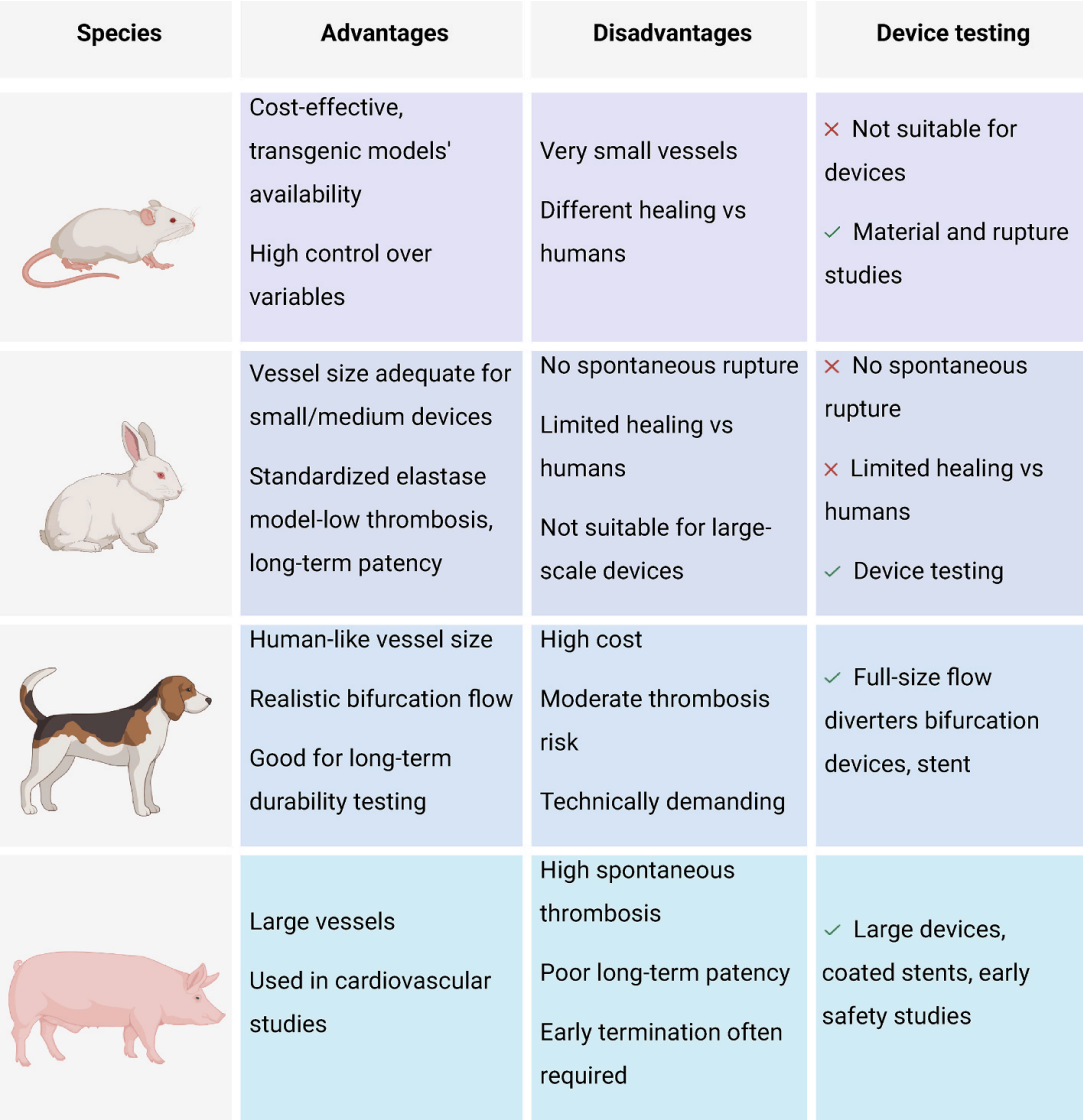
Comparative summary of preclinical animal models for aneurysm treatment strategies.

While specialized models like rat tail aneurysms provide isolated mechanistic insights, evaluating functional efficacy, such as the actual ability to occlude the aneurysm, remains a major hurdle in rodents [132,133]. Some studies have successfully created sidewall aneurysms in rodents to deploy flow diverters, but these approaches introduce a confounding overlap between the acute tissue response to microsurgery and the subsequent healing driven by the device, potentially altering the results [132,134]. Therefore, rodents are reserved for initial material biocompatibility and *in vivo* degradation kinetics rather than device efficacy testing [77,134].

The evaluation of full-scale device performance requires a transition to large animal models that can accommodate actual clinical dimensions. Canine and swine models, with vessel calibers closer to human arteries, are better suited for testing full-scale or long-term devices, although they are more expensive and have additional ethical constraints [134]. Swine models offer significant advantages due to their close similarities to human vascular physiology and coagulation systems, making them widely accepted in cardiovascular research. However, conventional pig models are suboptimal for chronic neurovascular studies due to their rapid growth rate, because significant vessel enlargement occurs within approximately four weeks, compromising the long-term evaluation of flow diverter apposition and degradation. The minipig model represents a highly effective alternative to mitigate this issue. Minipigs maintain a stable vessel size throughout the experimental period, allowing reliable long-term investigations up to twelve months to assess chronic vascular responses, intimal remodeling, and degradation control. Despite these advantages, very few studies have fully established minipig models for neurovascular flow diversion, meaning that the rabbit elastase model and the canine bifurcation aneurysm model remain the most established and valuable options for the functional assessment of aneurysmal devices [135]. The rabbit elastase model is particularly valuable because it features human-like aneurysm similarities, such as an attenuated elastic lamina and a high concentration of smooth muscle cells, and exhibits low thrombosis rates with long-term patency, mimicking unruptured human aneurysm. Thus, the canine bifurcation and rabbit elastase models are the gold standards for evaluating flow diverter efficacy, hemodynamic remodeling, and aneurysm occlusion.

Despite these benefits, differences in flow patterns, vessel caliber, and endothelialization rates influence the interpretation of preclinical results, particularly for bioabsorbable materials where degradation kinetics and endothelialization are tightly coupled. To provide clear preclinical evaluation standards toward clinical application, specific tasks must be distributed across these specialized systems in a structured sequence. Initial blood flow models, utilizing *in vitro* loops or *ex vivo* cadaveric loops, should be employed as an intermediate preparatory phase to optimize deployment techniques, verify wall apposition in straight versus curved configurations, and assess acute thrombogenicity under controlled flow conditions without ethical constraints. This phase leads directly to the small animal screening, which is dedicated to high-throughput assessment of material biocompatibility, local inflammatory cascades, and early polymer or metal degradation kinetics under a physiological environment. Subsequently, large animal chronic models evaluate long-term device efficacy, progressive aneurysm occlusion, and late neointimal coverage, while simultaneously monitoring framework degradation rates and structural integrity. Neurotoxicity assessments and behavioral observations are integrated through daily neurological scoring to exclude functional deficits, paired with postmortem histological analysis of the surrounding brain tissue to rule out parenchymal injury or reactive gliosis. Finally, crucial evaluations for bioresorbable devices screen the downstream cerebral microcirculation for potential embolic fragments from degradation, utilizing analytical techniques to detect cerebral element accumulation or polymer metabolites in target organs.

Collectively, these models provide complementary insights into different aspects of aneurysm healing, yet the lack of standardized methodologies and reporting metrics continues to limit cross-study comparability and translational predictability [134].

The sample size is also important in animal studies to prevent inconclusive results, because animal responses to intervention may be more heterogeneous than in humans. In effect, the current state of animal models for flow diverter research increases the number of animal studies needed to judge the clinical feasibility of new designs. This not only increases developmental timelines and costs but also conflicts with the 3Rs in biomedical research [136]. Studies using *ex vivo* setups or post-mortem tissue offer an alternative to some animal experiments, as well as 3D-printed and hydrogel-based approaches mimicking intracranial aneurysms *in vitro* [137,138].

### 3.3 Translational framework for preclinical device evaluation

The biomaterial, mechanical, and biological hurdles identified in the previous sections define the constraints for bioabsorbable flow diverter development. To address these challenges with a systematic approach, we propose a biomaterial-driven design framework. This methodology establishes practically applicable design criteria by linking fundamental material properties to performance requirements throughout the preclinical pipeline. As reported in Table 4, this framework maps study objectives onto performance targets, establishing the governing principles to mitigate these hurdles at each phase of the translational process.

**Table 4:** Biomaterial-driven design and validation criteria for bioabsorbable flow diverters.

Evaluation stage	Study objective	Experimental endpoints	Biomaterial-driven criteria
Material selection	Selection and sizing of wires or filaments	Tensile properties.	Selected material and strut dimensions match the targeted degradation window.
Manufacturing	Wire braiding, shape-setting, surface passivation, coating integration.	Braiding angle and metal coverage.	Uniform braided structure, structural integrity of any integrated surface layer; coatings applied without altering filament crossover mobility.
		Structural integrity post-thermal processing.	
		Coating thickness.	
Mechanical evaluation	Bench testing of the braided FD.	Radial force.	The braided structure demonstrates sufficient radial force while maintaining luminal patency and structural integrity during flexure in highly tortuous anatomies.
		Bending compliance and kink resistance.	
		Fatigue life under cyclic loading.	
*In vitro* degradation & biocompatibility assessment.	Degradation byproducts tracking and cellular safety.	Mass loss kinetics. pH variations and byproduct accumulation.	Scaffold degradation proceeds at a controlled rate without burst release or structure fragmentation toxic byproduct concentrations
		Cytotoxicity and hemocompatibility.	
Functional bench testing	Evaluation of delivery, deployment, and visibility.	Trackability.	Compatibility with 0.021–0.027″ microcatheter; radiopacity providing detectable X-ray attenuation.
		Expansion behavior.	
		Fluoroscopic visibility.	
*In vivo* validation	Translational safety and efficacy assessment in animal models.	Re-endothelialization and vascular integration.	The device achieves Grade 3 strut coverage before advanced wire fragmentation; complete aneurysm occlusion synchronized with host tissue remodeling without adverse systemic toxicity.
		Tissue remodeling and flow diverter degradation.	
		Downstream microvascular embolic and systemic toxicity screening.	

## 4 Conclusions and future directions

The development of bioabsorbable flow diverters requires shifting from single-material filaments toward composite architectures capable of predictable structural degradation over time. Evaluating the mechanical properties of bioabsorbable polymers and metals indicates that single-material designs fail to balance opposing clinical requirements. For instance, the low radial strength of bioabsorbable polymers necessitates increased strut cross-sections that compromise trackability and radial expansion mechanics. Conversely, magnesium wires exhibit rapid degradation rates when braided, causing premature mechanical failure before complete tissue encapsulation occurs. Consequently, composite architecture is necessary to overcome these material-specific limitations.

Integrating multiple materials into braided configurations requires optimizing surface-engineering workflows without altering device deployment behavior or mechanical flexibility. Applying thick coatings after braiding can lock filament crossover points and alter expansion behavior. However, coating individual wires prior to braiding is restricted by the high temperatures required for shape-setting. To resolve this trade-off, manufacturing must utilize deposition techniques applied post-shape-setting, restricting layer thickness to a sub-micron scale to preserve filament mobility at braid intersections.

This technical constraint applies directly to composite designs featuring an iron-manganese-nitrogen (FeMnN) alloy matrix over a molybdenum (Mo) core. While this core-shell architecture provides sufficient radial force at the micrometer scale to replicate the thin-wire profile of conventional devices and ensures integrated radiopacity without traditional non-resorbable markers, optimizing its acute hemocompatibility remains essential. To mitigate acute thrombogenicity without restricting device deployment performance, surface modifications should incorporate controlled passivation or partial conversion coatings. These treatments passivate the underlying FeMnN substrate and provide a stable template for an outer hemocompatible layer during the initial post-implantation phase, without interlocking the braided junctions.

Validating these advanced designs requires a tiered *in vivo* testing framework to address current preclinical safety and performance gaps. Initial screenings in murine models allow for the high-throughput evaluation of degradation byproducts and local tissue tolerance. Small-animal models offer logistically favorable costs, but their primary scientific value lies in the wide availability of specific molecular and cellular markers required to characterize the acute vascular response. Following baseline biocompatibility verification, studies can progress to large-animal models for functional validation. The rabbit elastase-induced aneurysm model remains the standard benchmark for evaluating acute flow stagnation and device-vessel interaction, whereas the minipig is the most appropriate model for chronic evaluation. Unlike juvenile domestic pigs, which exhibit rapid somatic vessel growth that confounds long-term degradation tracking, the stable vascular diameter of the minipig allows for the accurate assessment of degradation kinetics and the synchronization between mechanical resorption and neointimal healing.

Ultimately, the clinical translation of next-generation flow diverters depends on designing transient mechanical architectures where the reduction in radial stiffness coordinates precisely with the rate of progressive tissue remodeling. Aligning manufacturing processes with predictable degradation profiles ensures that the device acts as a temporary scaffold, sustaining hemodynamic loads until the remodeled vessel can independently support physiological blood pressure and wall shear stress.

## Ethics approval and consent to participate

The manuscript entitled “Defining biomaterial-driven design principles for bioabsorbable flow diverters: current state and perspectives”, co-authored by A. Di Lorenzo, A.F. Mohamed, B. Isella, D. Behme, A. Loewen, M. Vekemans, R. Schwab, T.C. Schmitz, S. Jockenhoevel, A. Kopp, submitted for evaluation to Bioactive Materials, is a literature review work, and thus no *in vivo* evaluations on animal model or clinical trials were performed in this scope. Thereby, our work does not fall into the incidence of ethical approvals and patient consents.

## CRediT authorship contribution statement

**Alessandra Di Lorenzo:** Conceptualization, Writing – original draft, Writing – review & editing. **Amr F. Mohamed:** Writing – review & editing. **Benedetta Isella:** Conceptualization, Writing – review & editing. **Daniel Behme:** Writing – review & editing. **Alexander Loewen:** Writing – review & editing. **Mark Vekemans:** Writing – review & editing. **Roland Schwab:** Writing – review & editing. **Tara C. Schmitz:** Writing – review & editing. **Stefan Jockenhoevel:** Writing – review & editing. **Alexander Kopp:** Funding acquisition, Writing – review & editing.

## Declaration of competing interest

The authors declare the following personal relationships which may be considered as potential competing interests: Alessandra Di Lorenzo, Benedetta Isella and Alexander Kopp are currently employed by Fibrothelium GmbH. Amr F. Mohamed, Mark Vekemans and Alexander Kopp are currently employed by Embocraft GmbH. Tara C. Schmitz is currently employed by Medical Magnesium GmbH. Alexander Kopp is currently employed by Meotec GmbH.
